# Recombinant expression of insoluble enzymes in *Escherichia coli*: a systematic review of experimental design and its manufacturing implications

**DOI:** 10.1186/s12934-021-01698-w

**Published:** 2021-10-30

**Authors:** Suraj Mital, Graham Christie, Duygu Dikicioglu

**Affiliations:** 1 Department of Chemical Engineering and Biotechnology, University of Cambridge, Cambridge, CB3 0AS UK; 2 Department of Biochemical Engineering, University College London, London, WC1E 6BT UK

**Keywords:** *Escherichia coli*, Recombinant industrial enzymes, Difficult to express enzymes, Inclusion bodies, Systems biology, Solubility

## Abstract

Recombinant enzyme expression in *Escherichia coli* is one of the most popular methods to produce bulk concentrations of protein product. However, this method is often limited by the inadvertent formation of inclusion bodies. Our analysis systematically reviews literature from 2010 to 2021 and details the methods and strategies researchers have utilized for expression of difficult to express (DtE), industrially relevant recombinant enzymes in *E. coli* expression strains. Our review identifies an absence of a coherent strategy with disparate practices being used to promote solubility. We discuss the potential to approach recombinant expression systematically, with the aid of modern bioinformatics, modelling, and ‘omics’ based systems-level analysis techniques to provide a structured, holistic approach. Our analysis also identifies potential gaps in the methods used to report metadata in publications and the impact on the reproducibility and growth of the research in this field.

**Supplementary Information:**

The online version contains supplementary material available at 10.1186/s12934-021-01698-w.

## Background

**Fig. 1:**
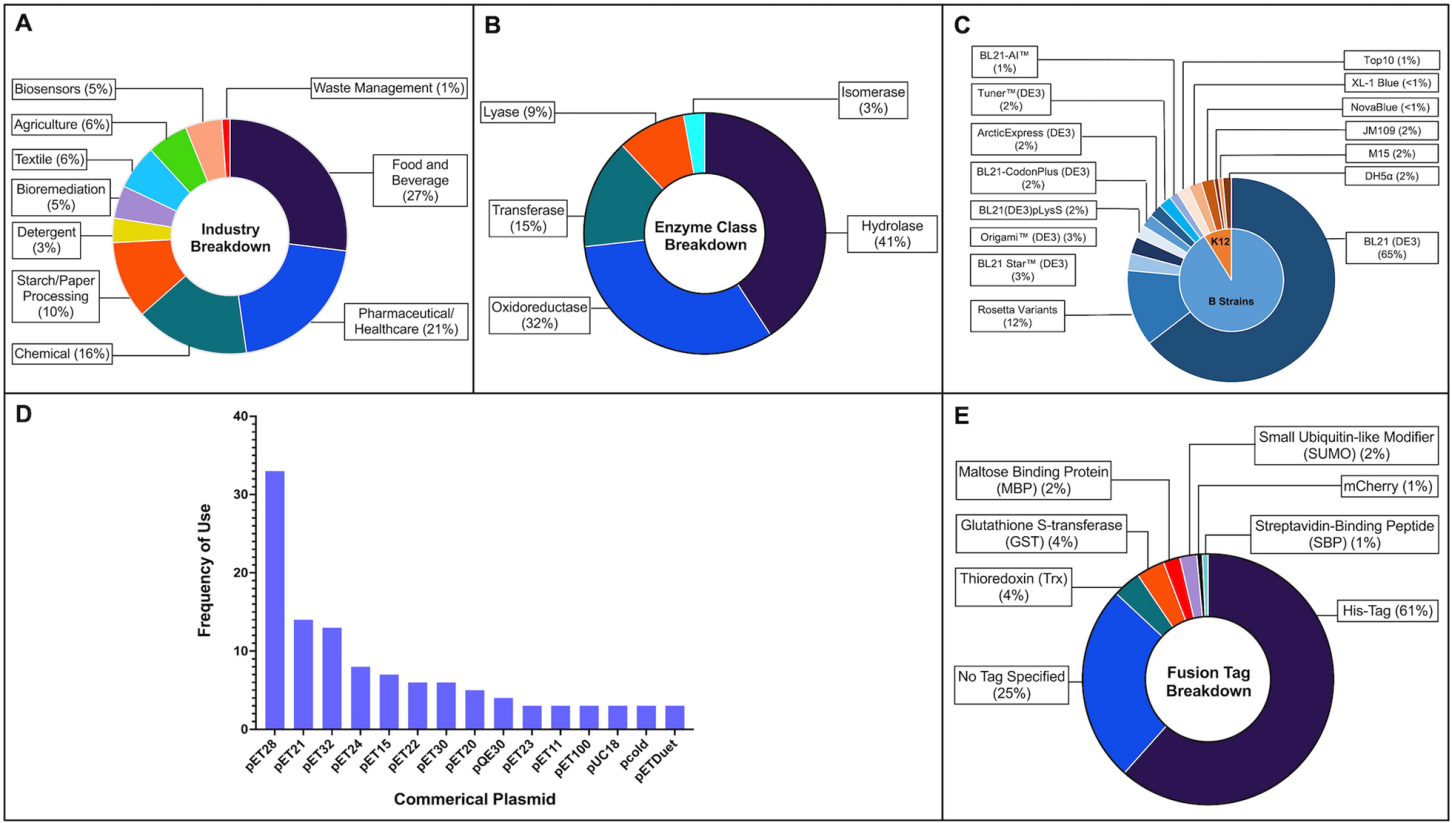
Trends in the selection of experimental design parameters for the literature surveyed in recombinant production of difficult to express (DtE) enzymes and industrially relevant enzymes in *E. coli* (year coverage: 2010–2020). **A** Breakdown of the industries in which DtE enzymes were most commonly employed or demanded. **B** Breakdown of the most common enzyme classes that DtE enzymes are affiliated to. **C** Breakdown of the most utilized commercial *E. coli* expression strains and their modified versions. **D** Frequency of plasmids (vector) used in different experimental designs. **E** Breakdown of the most utilized fusion tags in recombinant vector designs

Enzymes serve a wide range of biocatalytic purposes across multiple key industrial sectors; our observation shows the food and beverage, pharmaceutical/healthcare, chemical, starch and paper processing, detergent, bioremediation, textile, agriculture, biosensor, and waste management industries have the highest usage (Fig. 1A). The total biocatalysis market is a rapidly growing sector of industrial biotechnology with an estimated global market value projected to reach $10 billion by 2024 [1]. A review of the literature demonstrates a growing amount of research dedicated to discovering, isolating, and characterizing novel enzymes; this research is driven by a demand for enzymes that can replace current catalysts that show limited functional stability at specific operational conditions such as increased temperature or pH. Enzymes furthermore serve as tools to lessen the environmental impact of chemical processes traditionally driven by inorganic catalysts leading to ‘greener’ manufacturing [2].

Heterologous expression of a recombinant product is the preferred strategy when sufficient quantities of an enzyme of interest cannot be achieved in the native host organism. This method provides an efficient and economically favorable method to produce high quantities of recombinant protein in a relatively short amount of time. The industrial manufacturing sectors often adopt *E. coli* as a heterologous host for protein expression to facilitate rapid product. A common, challenging caveat to this expression method is the high likelihood of generating inclusion bodies due to protein misfolding. Inclusion bodies are aggregated masses of misfolded or partially folded peptide chains that can result from a variety of factors including but not limited to: when the rate of protein synthesis in vivo surpasses the capabilities of the cell, lack of eukaryotic chaperones for specific proteins, reduced cytosol environment, and limited post-translational machinery [3]; this is often the case when overexpressing a protein product in a recombinant expression system [4]; this misfolded state can be inhibitory to the biocatalytic capability of the enzyme and as a result, solubility is a property highly valued in the manufacturing supply chain. Over the past few decades, protocols have been modified by introducing different experimental design strategies to instigate the production of soluble products. These strategies explore variations in regulatory sequences (promoters), plasmid backbones, strains of *E. coli*, fusion partners employed, incubation temperatures, medium components, chaperone proteins, and inducer concentrations to name a few common variables.

In the event that these strategies prove unsuccessful, an extra refolding/renaturation and purification step is often necessary to generate a soluble, functional enzyme [5]. However, stepwise protocol of producing inclusion bodies and subsequent solubilization has proven to be a viable strategy to generate a higher volume of product. Recombinant protein within inclusion bodies has been found to occupy 30–40% of the total cellular proteins [6]; furthermore, inclusion bodies can be comprised of up to 90% pure recombinant protein [7].

A caveat to the solubilization methodology is that it is not a one size fit’s all strategy and often requires case-by-case protocols to be developed that cannot be widely applied all protein types. For instance, multi-copper laccases from four distinct organisms: *Bacillus* sp. HR03 [8], *Geobacillus* sp. JS12 [9], strains of *Yersinia enterocolitica* [10], and *Bacillus subtilis* strain R5 [11], though similar, prove to have unique purification protocols in each respective study. Furthermore, a loss in the secondary structure after exposure to strong denaturants can lead to a reduction in the overall bioactivity of the nascent protein [12]. The case-by-case specific nature of solubilization does not guarantee a final protocol fit for industrial use. Solubilization was shown to result in a reduced final recovery, from 50% or less of bioactive product in some cases [13] to no biologically active product in other examples [14]. At bench-scale, this is a small price to pay to generate protein that is appropriate for structural and or functional characterization. However, at industrial scales, the overarching costs associated with capacity underuse combined with operational costs including but not limited to consumables, utilities, and personnel time in addition to extensive protein quality control often render this option infeasible.

Current economic analysis associated with industrial scale manufacturing and downstream processing is limited in this field; there is a need for an updated techno-economic analysis of the processes discussed previously to reflect their current costs for the industrial biotechnology sector. Depending on the method utilized and the scale of operation; in 2011, the direct fixed costs and labor associated with this additional treatment was reported to add up to $8.2 million to implement and operate inclusion body solubilization equipment for individual companies [15], however these costs may be higher today. As a result, it is highly desirable to produce an enzyme of interest in a soluble state from the start to achieve cost-effective production.

### Scope of this review

A survey of the literature in this field from the past decade has revealed no standardized method developed to promote solubility for enzymes expressed through recombinant technology. This review identifies trends in the experimental design for recombinant expression studies, in the industrial biotechnology sector, that ultimately generated inclusion bodies in *E. coli*. Our analysis identified which methods or tools, if any, were employed in designing the recombinant expression system and the impact on the mitigation of inclusion bodies. Our goal was to highlight the factors/strategies researchers tend to prioritize and provide a measure for their popularity implicated by the frequency of their use. Our analysis was focused on work published in the field of industrial biotechnology since 2010. Manufacturing of numerous recombinant protein products, including those of biopharmaceutical use such as growth factors, antibodies, or cytokines had historically been in the remit of recombinant protein production by *E*. *coli*. Several well-cited reviews exist on the subject, which address the challenges associated with using *E. coli* for these purposes [16–18]. Over the past decade, Chinese Hamster Ovary (CHO) cells have increasingly dominated the manufacturing process as hosts for expressing protein-based biologics, therapeutics, and other relevant eukaryotic proteins. CHO cell-based systems are currently used to manufacture up to 84% [19] of approved biopharmaceuticals as opposed to 30% in *E. coli* [16]. This was primarily following the efforts towards the sequencing the CHO genome and its subsequent publication in 2011 [20]. Nevertheless *E*. *coli* has remained the standard workhorse for industrial biotechnology applications. Our analysis, therefore, focused on enzymes of prokaryotic origin and their plasmid-based expression in *E. coli*. These enzymes are industrially valuable due to their sustained performance in non-conventional niche environments owing to the wide distribution prokaryotes in adverse or unique habitats [21]. This reallocation of dominant sector preference for specific hosts in the manufacturing of different types of proteins has necessitated that even more attention needed to be paid to the improvement of host strains and expression systems of *E*. *coli* tailored to specialized applications. However, we still fail to see any systematic effort to streamline the development of efficient expression systems that overcome the insolubility of enzymatic proteins. The analysis conducted in this systematic review can serve this purpose and act as a starting point for future experimentation in the field.

### Identification of studies and selection

Databases such as NCBI PubMed and Clarivate Web of Science (WoS) provide a vast number of examples of scholarly literature that demonstrate the widespread prevalence of inclusion body formation. When used together for both databases, the search terms ‘recombinant’, ‘enzyme’, ‘inclusion bodies’, ‘*E. coli*’ and ‘*Escherichia coli*’ yielded 1891 publications, of which 659 were from the past decade (access June 2021). The omission of ‘*Escherichia coli*’ from these key words adds only 678 additional results indicating that *E. coli* continues to serve as the standard production workhorse and is discussed in 64% of recent recombinant enzyme expression work in this field.

**Fig. 2:**
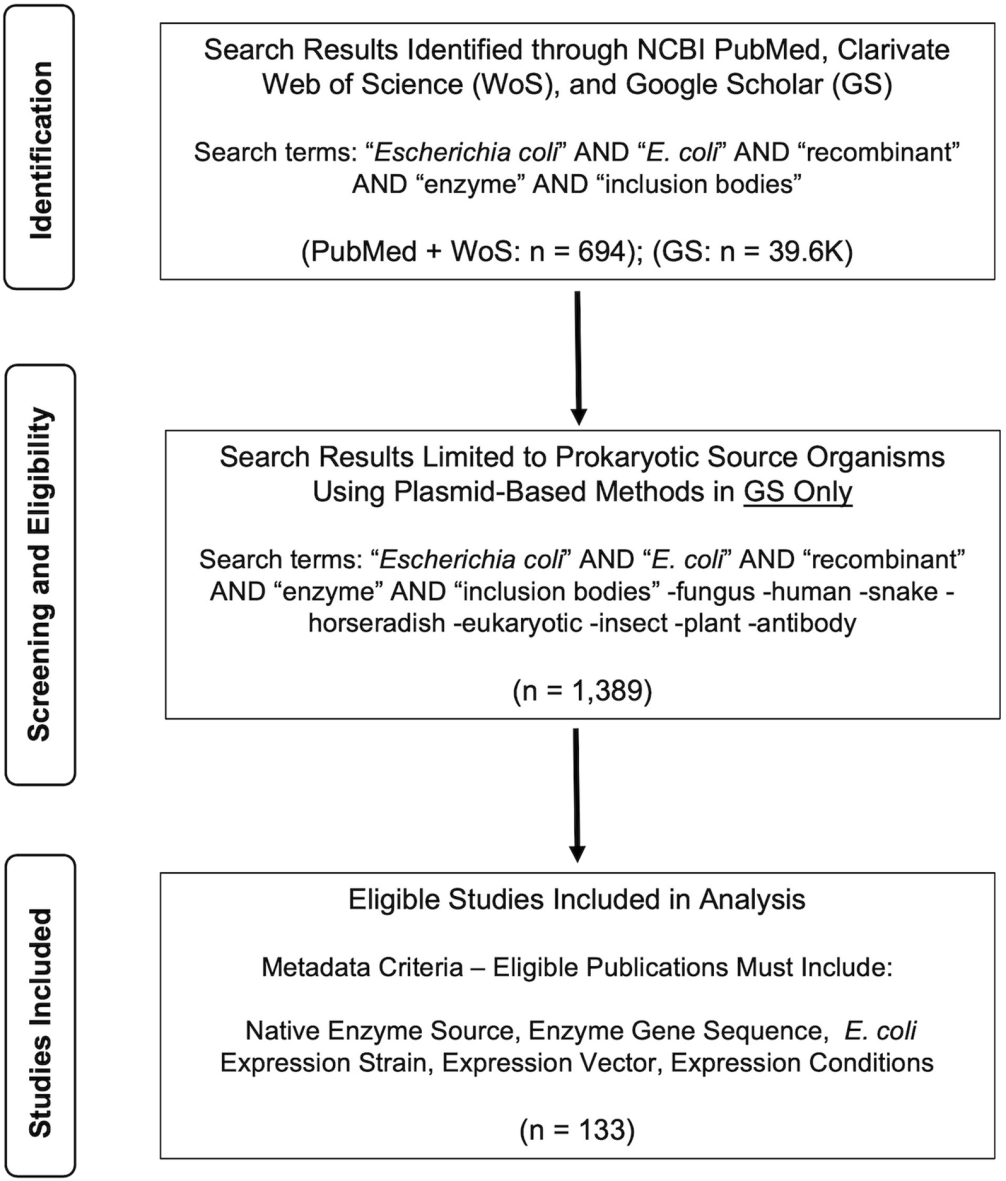
Study selection process. **A** Identification of total search results on NCBI PubMed, Clarivate Web of Science, and Google Scholar for key search terms. **B** Screening of total search results to narrow focus to publications with a focus on prokaryotic enzymes and plasmid-based expression methods. **C** Further screening based on metadata parameters

Publications for this review were sourced from NCBI PubMed and WoS databases, in addition to Google Scholar. Relevant articles were published between 1 January 2010 and 31 June 2021. Search terms included those for *Escherichia coli* (including the terms ‘*Escherichia coli*’ and ‘*E. coli*’), ‘recombinant’, ‘enzyme’, and ‘inclusion bodies’, generating a total of 501 results in PubMed, 193 result in WoS and 39.6 K search results in Google Scholar (Fig. 2). The difference in the number of search results generated between PubMed/WoS versus Google Scholar is due to the algorithm employed. A large proportion of the search results on Google Scholar have been found to be ‘grey literature’—a term that encompasses books, book chapters, patents, theses, non-peer reviewed research, and/or ambiguous citations that do not fall within a specific category, in addition to duplicate search results [22]. This volume of grey literature accounts for the discrepancy of search results generated.

These papers were manually curated within the search results for PubMed and WoS. Logical operators were used to remove irrelevant search results from the large number of results returned in Google Scholar specifically leaving 730 results; this curation process is highlighted in Fig. 2. The metadata reported by a given study is required for future reproducibility. Therefore, our literature search for this review was limited to those studies that explicitly reported: (i) the native source of the enzyme of interest, (ii) gene sequence, (iii) the *E. coli* expression strain, (iv) the expression vector backbone, and (v) expression conditions (i.e. expression temperature, inducer concentration, cell density). These details are deemed essential for reproducibility in this field. 21% of the publications, which were initially thought to be relevant to the scope of this review, were omitted from further investigation due to the lack of at least one of the aforementioned details (Fig. 2). In total 133 publications that expressed 140 enzymes were selected between 2010 and 2021; some publications highlighted multiple enzymes leading to a higher number of enzymes compared to publications. A full list of this literature can be found in Additional file 1. Interestingly, all enzymes were identified to be relevant within industry or for direct commercial sale, although this aspect was not explicitly mentioned in their respective publications.

We observe that the food and beverage, pharmaceutical, and chemical industries showed the largest demand for bulk biocatalyst manufacturing and had the greatest variety of enzymes employed in their processes during the period of investigation (Fig. 1A). Hydrolases (41%) and oxidoreductases (32%) were the most prevalent classes of enzymes studied in the publications investigated within the scope of this review, followed by transferases (15%), lyases (9%), and isomerases (3%); none of the enzymes recombinantly expressed in these studies belonged to the class of ligases (Fig. 1B). This breakdown suggests that hydrolases and oxidoreductases are of substantial industrial interest, however it is difficult to interpret from this data whether these classes have a higher propensity to form inclusion bodies in comparison to other enzyme classes.

### Data reporting and limitations

The narrative of published research targets a selective audience in its cognate field of work, for whom each detail regarding the experimental design may not necessarily be of interest. Our analysis identified an absence of methodical organization, or a system to report experimental design details. The aforementioned details were reported in all 133 studies, however the curation process for this review required a comprehensive search of the introduction, methods, and supplementary text of each publication. This method of reporting metadata is inefficient, limiting reproducibility by the scientific community post-publication. Research narratives are centered around their principal objective; this objective guides the details emphasized and their priority within the text. For example, in characterization studies, where the primary goal was to report on enzyme structure, less emphasis may be placed on the expression conditions used to produce of the enzyme of interest. This bias was observed in the past in The Protein Data Bank (PDB), the major repository for protein structural datasets. Zhou et al. reported that expression host information for recombinant studies were omitted from 62 (12%) of the PDB study examples they selected for their analysis [23]. In another case, Magnan et al. identified that the PDB and the SwissProt databases were observed to report on the solubility of recombinant proteins but neglect to include the experimental conditions that were used; this was critiqued as the information provided by the retrieved datasets retained inconsistencies rendering it ineffective for modeling purposes [24]. The omission of such details can often hamper follow-up work by scientists in other fields.

One other aspect of this work worth noting was only one out of the 133 studies shortlisted was conducted through a collaborative partnership with industry [25]. The remaining studies were led by either academic institutions or government agencies. This underrepresentation of industrial contribution does not truly reflect the economic challenges inclusion body formation poses on commercial manufacturing [26]. However, this contributes a bias to the information currently available in the public domain. The issues highlighted above demonstrate that a degree of implicit bias currently limits our understanding to facilitate reproducible research. The evaluation discussed below should be considered in light of these limitations.

### Enzyme discovery and in vitro expression

Recently, the process of enzyme discovery has been led by metagenomics and genome mining from environmental microbiome samples [27–29]. Screening a range of habitats with varying physical environmental conditions have led researchers to uncover organisms with evolutionarily adapted tolerance to these conditions. Higher temperatures, for example, in the case of *Thermus thermophilus* HB9 [30], cold temperatures, in the case of *Shewanella arctica* [31], or elevated salt concentrations in the case of *Halobacterium salinarum* [32] are examples of adapted bacteria. Researchers have observed that enzymes isolated from these unique prokaryotic hosts share a similar tolerance to environmental conditions in vitro and therefore have use in industrial manufacturing conditions that are typically harsh in comparison to the growth environment of many organisms. In other instances, in vitro directed evolution through mutagenesis or computationally driven protein engineering can serve as alternative methods to develop stable or functionally novel enzymes within a laboratory setting [33]. Directed evolution, for example, was used to expand the substrate range of *Rhodococcus* phenylalanine dehydrogenase for the highly enantioselective reductive amination of ketones to amines [34].

While the enzymes from extremophiles are of industrial interest, extremophilic bacteria, for example, are often difficult to culture in a laboratory setting due to challenges associated with providing culture conditions that adequately mimic their native environment [35–43]. These additional considerations create a non-standard set of culture conditions. Moreover, enzymes isolated from organisms found in complex environmental samples pose additional challenges as optimal culture conditions for many organisms are typically not well*-*understood. Such a challenge was reported for a strain of *Aeromonas hydrophila* isolated from sludge samples collected from textile wastewater treatment plants; the complex chemical composition of this sludge rendered it difficult to determine a suitable, replicable medium formulation to support growth of the organism in a laboratory setting [44]. Therefore, the process of generating suitable high quantities of active enzyme from a novel isolated prokaryotic source is not always a straightforward task.

In other cases, certain enzymes have been identified as being toxic to host cells when overexpressed at high levels. Microbial transglutaminase (MTGase) monomers from *Streptoverticillium mobaraensis*, for example, were shown to have a high tendency to cross-link and oligomerize during intracellular expression [25]. MTGase was also observed to be expressed at low abundance in *S. mobaraensis*—at concentrations inadequate for subsequent experimental work—which is an issue frequently observed in similar studies [45–49]. Additionally, enzyme purification methods from native host sources often require a combination of methods such as salt precipitation and/or a series of chromatographic separations to deliver a product with sufficient purity. However, these methods are often difficult and economically challenging at scale, particularly when purifying enzymes expressed in limited quantities [50, 51].

## Current experimental design practices

### Expression strains—K12 and B strains

A majority of the available *E. coli* expression hosts in use for recombinant protein expression typically fall under two categories: K12 or B derivative strains. BL21 (DE3) is one of the most common B strains preferred for recombinant expression; often, it is preferentially selected over its K12 counterparts as an ideal recombinant expression host due to several key advantages. BL21 (DE3) is deficient in Lon and OmpT proteases, thus providing a layer of protection to misfolded proteins that would normally be targets for degradation [52]. A short doubling time of approximately 20 min coupled with rapid protein synthesis via the T7 expression system generates a stable protein product at high titers [53]. K12’s longer growth times and predisposition to produce acetate generates lower biomass in comparison to BL21 (DE3) [54].

Our analysis observed B strains were the most widely employed for enzyme expression in 88% of the total cases. Among those, BL21 (DE3) was selected as the primary expression host in 65% of our reference cases (Fig. 1C). The remaining 12% utilized K12 derivates. Commercially available K12 strains used include JM109 [25, 51, 55], DH5α [30, 56], NovaBlue [57], XL1 Blue [58], M15 [31, 59, 60], and Top10 [61, 62]; alternatively non-commercial K12 strains W3110 [63] and MG1655 [62, 64] were used in a limited number of cases. K12 strains serve as useful tools when plasmid instability is encountered resulting in plasmid loss from the host [54]; this may explain its use in limited cases for expression. Beyond protein expression, K12 strains are also mentioned as tools plasmid propagation and cloning. For example, Vadala et al. [65] propagated their pET21a vector in DH5α however their eventual expression host was BL21 (DE3). A breakdown of commercial strains used, and their applications can be found in Table 1; a full list of expression strains used can be found in Additional file 1: Table S1.

**Table 1:** Commercial *E. coli* expression strains employed for the production of DtE enzymes (2010–2021)

Expressed industrial enzyme example	*E. coli* expression strain	Commercial supplier	Benefit	References
Bis-γ-glutamylcystine	ArcticExpress (DE3) (B)	Agilent Technologies, Inc	Expression at low temperatures with active molecular chaperones. Promotes ideal folding under low temperature conditions, increasing solubility	[183]
Malto-oligosyltrehalose trehalohydrolase	Origami™ B (DE3) (B)	Merck KGaA	Expression of proteins rich in in disulphide bonds. Promotes cytoplasmic disulphide bond formation	[66]
Phenol hydroxylase component 2	BL21-CodonPlus (DE3) (B)	Agilent Technologies, Inc	Expression of proteins rich in AGA/AGG, AUA, and CUA. Promote correct synthesis and folding for proteins with rare codons in *E*. *coli*	[184]
Flavin reductase	BL21(DE3)pLysS (B)	Various Suppliers (Thermo Fisher Scientific, Promega Corporation, Merck KGaA)	Lower background expression, useful for toxic proteins. Allows greater control over expression	[73]
Arylamine *N*-acetyltransferase				[14]
Beta-glucosidase	One Shot™ BL21 Star™ (DE3) (B)	Thermo Fisher Scientific	High expression of non-toxic proteins. Promotes high expression of protein product	[74]
*N*-Acyl-d-glucosamine 2-epimerase	Tuner™(DE3) (B)	Merck KGaA	Allows slower protein synthesis to promote solubility using adjustable inducer concentrations	[71]
Sphingomyelinase	Rosetta/Rosetta 2 (DE3) (B)	Merck KGaA	Promote correct synthesis and folding for proteins (i.e. eukaryotic proteins) with rare codons (AGA, AGG, AUA, CUA, GGA, CCC, and CGG) in *E*. *coli*	[79]
Lysine 6-Dehydrogenase	Rosetta-gami™ (DE3) (B)	Merck KGaA	Alleviates codon bias and enhances disulphide bond formation	[83]
2-hydroxyethyl-phosphonate methyltransferase	Rosetta™ 2 (DE3)pLysS (B)	Merck KGaA	Alleviates codon bias and lowers background expression, useful for toxic proteins	[84]
Tryptophan-2-C-methyltransferase	RosettaBlue™(DE3) pLysS (B)	Merck KGaA	Alleviates codon bias and lowers background expression, useful for toxic proteins. High transformation efficiency	[78]
Fructose-1,6-bisphosphate aldolase	DH5α (K12)	Various Suppliers (Thermo Fisher Scientific, New England Biolabs, Gold Biotechnology Ltd.)	Generally, a strain used for cloning and blue/white screening. (recA) mutation allows for better insert stability	[30]
Chitobiase	M15 (K12)	Qiagen	Generally used in conjunction with plasmid (pQE) found within a expression kit from Qiagen	[59]
Cellobiose phosphorylase	JM109 (K12)	Promega Corporation	Generally, a strain used for cloning and blue/white screening	[55]
Quinoprotein glucose dehydrogenase B	NovaBlue (DE3) (K12)	Merck KGaA	Generally, a strain used for cloning and blue/white screening	[57]
Fuculose-1-phosphate aldolase	XL-1 Blue (K12)	Promega Corporation	Generally, a strain used for cloning and blue/white screening	[58]
Trehalose transferase	TOP10 (K12)	Thermo Fisher Scientific	Generally, a strain used for cloning and plasmid propagation	[61]

### Specialized *E. coli* strains—emerging tools for protein expression

The challenging nature of DtE enzymes has led to the adoption of alternative hosts to improve performance, with several companies developing strains that researchers are opting for in favor of traditional strains. These BL21 (DE3) variants were the strains of choice in 30% of the selected expression studies. Industrial manufacturers have evolved the efficiency of their strains by producing variants capable of reducing the common causes of aggregation that manifest during overexpression (Table 1). These strains are marketed to have superior performance that are well-suited to mitigating issues concerning:

i. High disulfide bond formation—BL21 Origami B [66]
ii. Codon Bias BL21 CodonPlus [67]; BL21 Rosetta
iii. Temperature instability—BL21 ArcticExpress [14, 31]
iv. Toxic proteins—BL21 AI [68, 69], BL21 Tuner [70–72].
v. Low expression yield—BL21 pLysS [14, 73] and BL21 Star [74, 75].

The reason behind the selection of a specific strain is not overtly mentioned in the text in a majority of the cases investigated, but rather it may be inferred from the broader context of the study. The researchers’ selection could also be led by previous experience or through justification using structural bioinformatics to gain insight as to how a protein of interest may behave in vivo. Many options exist for specific expression issues, and therefore these strains are adopted on a case-by-case basis. BL21 Origami (DE3), for example, was identified to be an ideal starting strain for the expression of maltooligosyl trehalose trehalohydrolase (MTHase) as the protein’s structure and, in turn, enzyme function was impacted by disulfide linkages [66]. Likewise, pLysS strains of BL21 showed promise to produce soluble levels of toxic proteins such as certain metalloproteins [76]. In other instances, the use of strains such as ArcticExpress are motivated by promoting solubility through facilitating expression at low temperatures, which is a widely accepted strategy that is implemented to control the rate of synthesis.

In addition to the strains mentioned above, Rosetta, which has many variants available (Table 1), has a growing popularity as an *E. coli* host for DtE bacterial enzymes, engineered to have an increased tRNA supply for such codons as AUA, AGG, AGA, CUA, CCC, GGA, which are less abundant in *E. coli* [77–80]. Originally this strain was utilized for the expression of complex eukaryotic proteins [81]. However, it is often thought that tRNA limitations can factor into the formation of inclusion bodies [82]. Other studies utilized modified strains of Rosetta such as Rosetta-gami-pLysS [83] and Rosetta pLysS [84], which bring additional benefits of the Origami and pLysS systems together with the enhanced tRNA supply of Rosetta (Table 1). Other enhanced expression strains, include BL21 Lemo21 [85] for proteins potentially displaying toxicity effects such as membrane proteins, currently exist in the market but are not included in Table 1 since these tools were not used as expression hosts for the studies selected within the scope of this review.

Specialized BL21 (DE3) *E. coli* strains were utilized in only 30% of the studied surveyed here and in a large majority of cases, these specialized strains had little effect in the context of their respective studies. It often appears that the success of such strains varies on a case-by-case basis based on the properties of the protein and the additional expression parameters used. Soluble expression of MTHase from *Sulfolobus acidocaldarius* was improved by 40% when this protein was expressed in *E. coli* Origami (DE3) as opposed to BL21 (DE3); this enzyme’s structure is dependent on disulfide linkages for proper folding. This study noticed the combination of *E. coli* Origami (DE3) in addition to using thioredoxin (Trx) as a fusion partner on pET32a improved the folding propensity of MTHase [66]. Likewise, the use of maltose binding protein (MBP) coupled with rare tRNAs found in *E. coli* Rosetta (DE3) improved the expression of prenyltransferase (NovQ) by up to 50% [86].

In each of the cases described above, solubility was improved in combination with another experimental factor. It is therefore difficult to discern what the true impact of the specialized strains on the final recombinant product. It is possible that a combination of such strain and experimental design parameter combinations impact the final end-product. BL21 CodonPlus (DE3)-RIL on its own demonstrated no improvement for *Bacillus subtilis* strain R5 laccase, however a 30% improvement was observed when the expression temperature was lowered from 37 to 17 °C [11]. We require larger amounts of aggregated metadata to draw such accurate conclusions.

Despite their success in previous studies, topoisomerase I from *Mycobacterium tuberculosis* when expressed in BL21 Arctic Express [87] or DAHP synthase from *M. tuberculosis* when expressed in BL21 Rosettagami [88], specialized strains remain an underutilized potential solution to address difficulties around the formation of inclusion bodies during heterologous expression of DtE enzymes (Fig. 1C). We observe that no specific specialized strain was preferred over the others; the variants of Rosetta, when treated as group, were the second most prevalent strain after BL21 (DE3) accounting for 13%.

It is possible that specialized strains have found limited use to date as they are a relatively recent development in comparison to the first reports of BL21 (DE3); Studier and Moffatt introduced BL21 (DE3) in 1986 [89], whereas the specialized strains were developed years later; BL21 Origami in 2001 [90], BL21 Star in 2002 [91], and Rosetta-gami B in 2005 [92], offering a possible explanation for their current underutilization. One other possible reason could be that although modified strains have been designed to alleviate the impact of specific challenges persistently faced in the expression of DtE enzymes, they may have limited application areas outside the scope of their tailored use. This coupled with the additional costs to purchase each individual strain make this process economically unviable for many laboratories. For these reasons, it is likely that long-established research environments prefer to opt for the tools (i.e. expression strains) that are readily available within their existing practice.

In 58% of the papers we reviewed, the research narrative did not overtly mention experimental factors taken into consideration to promote solubility and rather focused on their downstream solubilization methodology. From our observation, only the approaches that led to the final protein of interest were detailed in each paper, while other unsuccessful attempts may have been omitted.

### Plasmid considerations for DtE enzyme expression

Whereas the previous section focused on different strains available for expression experiments there are actually far more variants of plasmid vectors employed in the past decade for the expression of difficult and problematic enzymes in *E. coli*. We observed a large demographic of vectors utilized throughout. pET vectors were by far the most commonly employed (in 66% of cases), and they were primarily utilized in conjunction with DE3 strains since the T7 RNA polymerase gene of DE3 is required for efficient synthesis of sequences downstream of T7/Lac hybrid promoters present in many pET plasmids [93]. pET28 was the most popular choice of vector, featuring in approximately 31% of the studies that utilized pET derivatives, followed by pET21 [8, 29, 36, 94–96], pET32 [97, 98], pET24 [99, 100], and pET15 [48, 77] (Fig. 1D).

The remaining 34% of plasmids did not fall into a singular group with high use and were uniquely employed in the study reporting them in varying frequencies. We observe that certain plasmids were used for specific purposes; a similar observation to that of the specialized strains discussed previously. The pCold(I–III) plasmid set was used for expression at cold temperatures i.e., whereby expression is induced by the cold-shock response [83, 101, 102]; this plasmid was used in conjunction with ArcticExpress (DE3) for suitable expression at temperatures below 13 °C. pACYC-Duet-1, with its two multiple cloning site locations, was utilized for the co-expression of native chaperones proteins from *Pyrococcus furiosus* as a strategy to promote proper folding of an α-amylase [103]. A full list of expression vectors used can be found in Additional file 1: Table S1.

Tight basal expression control of proteins appeared to be a key factor that was prevalent among the literature. In most instances, this control was managed through lower concentrations of inducer compounds such as 0.1–0.5 mM IPTG for inducible promoters such as T7/lac [6, 59, 65, 72, 104–106]. Soluble expression of *Bacillus acidopullulyticus* pullulanase was highly dependent on the control of basal gene expression that was only achieved in pET22b/pET28a harboring an inducible T7/lac promoter as opposed to pET20b that contained a constitutive T7 promoter region [104]. Tighter regulatory control was observed when a PHsh vector was used to moderately enhance the solubility of *Thermus thermophilus* HB27 pullulanase. PHsh contains a synthetic heat-shock promoter (Hsp); proteins synthesized on this vector system are regulated by the heat-shock transcription factor σ^32^. It was observed that *E. coli* JM10 reached a higher cell density and displayed a lower stress response in comparison to expression using T7/lac in BL21 (DE3). To contrast, however, solubility of *Mesorhizobium loti* carbonic anhydrase was enhanced using the J23100 constitutive promoter in combination with a N-terminal TrxA fusion on a pSUM backbone and co-expression of GroEL/ES; this improvement was in comparison with a similar experimental design with pET28a and pET32a using an inducible promoter system [107].

These examples represent a small fraction of the options available for expression plasmids, with variants available from different manufacturers. The vast number of vector-expression strain combinations allow for a range of different possibilities for researchers to customize their experimental designs. Customarily, commercial suppliers offer plasmids with different combinations of promotors, selection markers, multiple cloning sites, and fusion tags adding further to the myriad of combinations a researcher can ultimately choose to utilize in their designs. Discussion of factors contributing to the final design were extremely limited; this further contributes to the challenge of facilitating rational decision making for designing protein expression studies.

### Use of fusion tags in construct designs

77% of publications discussed the use of at least one fusion tag within their design; polyhistidine tags (His-tag) comprised 83% of all tags used. Fusion tags are essential tools for protein recovery as well as for the analytical quantification of products. Commercial plasmids often contain peptide sequences encoding tags that can be used for purification, act as reporter genes, or promote solubility. pSUMO/Champion™ pET SUMO Expression System are examples of plasmids engineered to contain a native SUMO tag to enhance the solubility of fused proteins [78, 108, 109]. A majority of pET vectors such as pET28, pET15, and pET21 contain incorporated His-tags, adjacent to multiple cloning sites, that can be used for downstream purification using immobilized metal affinity chromatography columns (see manufacturer’s manual, Novagen, accessed June 2021). This could explain why we observe a larger use of pET expression vectors and His-tags in these enzyme characterization studies. However, it is important to note that His-tags can be incorporated independently of features encoded on plasmids using specific primers encoding the His-tag sequence.

Apart from facilitating affinity purification [110–112], fusion tags can serve essential roles to enhance and promote the solubility of difficult to express enzyme constructs. In a small fraction of the studies reviewed here (16%) peptide tags such as thioredoxin (Trx) [66, 98, 113], glutathione S-transferase (GST) [114], small ubiquitin-related modifier (SUMO) [108], and maltose binding protein (MBP) [70] were used to promote solubility (Fig. 1E). These values were calculated based on whether the text mentioned the tag in their narrative. As mentioned previously pET32a, for example, contains a TrxA tag, however the number publications mentioning both pET32a and TrxA is not equally proportional. Therefore, the frequency of use for these fusion partner proteins may be higher in reality; it is difficult to interpret based on current metadata reporting practices.

The widespread use of the His-tag system suggested that bench-scale enzyme characterization studies prioritized producing an enzyme product regardless of its physicochemical state; we observe these publications mention protocols for refolding inclusion bodies back to their native state following downstream purification. However, the preventative steps considered to avoid the formation of inclusion bodies in the first place were not explicitly mentioned. Furthermore, in some cases production of inclusion bodies was a preferred strategy for ease of purification or as the only means to achieve large amounts of protein for characterization. However, as mentioned previously, this approach is not economically feasible for industrial scale manufacturing.

Suppliers such as Novagen provide tags within a majority of their constructs for fusion at the N-terminal (see manufacturer’s manual, Novagen, accessed June 2021). We observed a twofold stronger preference for N-terminal allocation of the His-tag than for C-terminal allocation in the reviewed literature [58, 104, 114–116]. C-terminal fusions were found in a limited number of cases [35, 40, 42, 51, 57, 117–119]. However, the discussion on the factors contributing to this predilection was limited; our analysis of the reviewed literature did not indicate a strong benefit received from either choice in terms of the physical condition of the final product. The choice of tag location is typically guided by an enzyme’s structure, as to not interfere with the active site during catalysis. Fusion partners attach additional peptide residues to the construct. This could, in some cases, increase the possibility of misfolding due to the increased size of the construct, although this was not explicitly acknowledged or studied in detail. Primarily, studies using His-tags did not mention downstream removal of the tag from the protein of interest; generally larger fusion partners such as MBP [86], TrxA [120], or SUMO [109] were removed via protease cleavage sites following purification.

Our analysis suggests that the adoption of fusion partners is mainly for purification and is not a widely utilized technique to promote solubility of DtE enzymes despite past successes [121, 122]. The few instances, mentioned previously, showed MBP and TrxA promoting solubility in conjunction with other experimental parameters like temperature, inducer concentration, and/or expression strain. However, it is difficult to discern whether these fusion partners have merit for a wider range of proteins. A full list of fusion tags used can be found in Additional file 1: Table S1.

## Role of systems biology in addressing the challenges around the formation of inclusion bodies

The physiological effects of expressing a recombinant enzyme in *E. coli* was infrequently considered in the literature that we have discussed until now. Heterologous protein expression in prokaryotic recombinant systems is not always a straightforward task following a clear and well-defined recipe. Biological processes are optimised for supporting the organism’s survival; overexpression of foreign enzyme products causes system-level responses in the transcriptome, metabolome, and proteome of *E. coli* as observed by dynamic changes introduced to gene expression [123]. Recombinant protein expression and the overproduction of a heterologous protein was reported to potentially create a large burden on the cell, consequently leading to stress [4]. Although *E*. *coli* cells are agile, they can only adapt to stress conditions, such as increased protein synthesis, to a certain limit. In such instances, metabolic resources normally dedicated to cell propagation would then need to be committed to the synthesis of a non-endogenous protein product. Additionally, the production of misfolded aggregates would lead to the accumulation of low-quality products that the cell would not be able to breakdown or fully refold back to their native state [123].

Within our analysis, the discussion of changes at the metabolomic or the proteomic level in response to overexpression was very limited. Understandably, this was not the intent nor the purpose of the research narrative in the evaluated studies. Research appears to take advantage of inclusion bodies; these act as sources of relatively pure, stable, and large protein deposits that can be easily isolated for refolding, as a means to end to generate the enzyme of interest [124]. However, it does beg the question whether this experimental design can be improved with a holistic, systems perspective to optimize protein synthesis, promote solubility and in turn reduce the need for additional downstream processing steps. Within this field of research, we find a growing number of alternative outlooks, led by omics-based techniques and bioinformatics, which could potentially be used to modify or evaluate experimental parameters to improve the expression of DtE enzymes. We will discuss these approaches in addition to the use of computational tools within our selected review papers in the subsequent sections.

### Role of bioinformatics and modelling within our literature survey

Among the selected literature, the use of bioinformatics and modelling tools for structural and functional characterization was discussed in 19% of cases. Primarily, computational methods we found to be used in the context of identifying uncharacterized gene clusters encoding enzymes from environmental samples such as a cold-active esterase from *Rhodococcus* sp. AW25M09 found in arctic ocean water [125] or a halotolerant lipase from *Marinobacter lipolyticus* found in the hypersaline regions of southern Spain [42]. Adaptation traits provide molecular biologists and industrial manufacturers additional tools capable of withstanding adverse conditions of temperature, pH, chemicals (i.e. co-solvents), or salinity for example [126]. In other instances, genome mining of sequenced organisms or related species highlighted uncharacterized variants of well-known enzymes such as sarcosine oxidase [127], or β-agarase [108].

Homologous sequence alignment tools, such as BLAST [128] were used to assess and compare the similarity of novel enzymes to known sequences found within the GenBank online database [129]. The conserved regions for a given enzyme were compared across species using alignment programs such as EMBL-EBI’s Clustal Omega [130–133] or other algorithms such as Needleman–Wunsch Global Align Nucleotide Sequence [68, 134]; these conserved domains, such as active site residues, assisted researchers to interpret and predict an enzyme’s function [135]. Beyond these methods, we observed the use of SWISS-MODEL [127, 135–137] and PyMOL [42, 138] for comparative 3D modelling of the evolutionary relationship between target proteins and the prediction of substrate-enzyme docking interactions. It is expected that this methodology for predictive structural modelling of new DtE enzymes will be highly influenced by innovations incorporating novel neural network architectures such as AlphaFold [139]. In a limited number of cases (5%) SignalP prediction server [140] was used to detect putative signal sequence motifs in the gene sequence of the novel enzyme and extrapolate the native subcellular localization the enzyme when expressed [108, 132, 141, 142].

### Peptide sequence-driven computational methods to predict solubility of DtE enzymes

Sequence-based analyses can highlight the folding patterns of proteins such as how surface residue patches can interact with their surrounding environment. Protein engineering research has observed that larger patches of positively charged residues and hydrophobic surface residues impact aggregation within proteins. A mutation in a single residue can tremendously impact the charge distribution of recombinant proteins and in turn increase solubility [143]. Furthermore, restricting the exposure of hydrophobic patches on a protein’s surface has been shown to increase the likelihood of producing a soluble protein in aqueous environments, depending on the ratio of hydrophobic to polar amino acids on its surface [144].

Sequence-based modelling tools, derived from the statistical solubility model of Wilkinson and Harrison [145, 146], such as PROSO II and SOLpro can help predict the solubility of a protein by its amino acid composition. These tools take sequence-specific factors including folding propensity, residue charge, cysteine fractions, and hydrophilicity, into consideration in their algorithms [24, 147]; often, these programs can be a preliminary resource to predict the folding patterns of a protein of interest. In the past, similar sequence-based prediction methods were used to evaluate plasmid design by ranking the choice of expression constructs with fusion carrier proteins such as NusA, GrpE, and thioredoxin bound to insoluble protein human interleukin-3 (hIL-3) in *E. coli* [148]. It was found that this method was successful in predicting the effectiveness of a given tag in promoting solubility when fused to hIL-3. In another study, Chan et al. applied a model-based approach to assess the cloning regions of six vector designs for the effect of varying the location of solubility fusion tags (Trx, MBP, NusA) and affinity tags such as the His-tag on the solubility of their product; their methodology presented a model to evaluate the design of plasmids for recombinant expression—validated by machine-learning based prediction tools [149]. Often, despite their potential, such modelling-based tools are still criticized for disregarding sequence-independent features such as growth temperatures, media conditions, inducer concentration, etc. that also play a role in the formation of inclusion bodies [150]. Furthermore, there is a need to validate such models through experimental methods. The sequence-based protein design algorithm—PROSS has already been validated by community-motivated efforts against a range of DtE proteins; it was found that 9 out of 14 target proteins showed improvement in heterologous expression under the experimental conditions designed by the prediction tool [151].

However, recent efforts on the recombinant expression of DtE enzymes in *E. coli* did not indicate bioinformatics were involved with experimental or amino acid sequence evaluation—despite the open-access to such tools. For example, our analysis did not observe a systematic consideration when selecting fusion partners in the design of an experiment, but the process was rather ad hoc, with decisions likely being made based on prior experience. Contrary to existing practices, computational sequence-based modelling tools may be useful to predict how a protein may be expressed based on the design of an expression vector in addition to guiding protein engineering. Design modifications can be made based on these predictive models on the road to promoting the solubility of a DtE enzyme. The use of these advanced technologies can expand our capabilities to systematically investigate aspects of protein biology and streamline our decisions for future experimentation.

### Codon bias and peptide sequence as modulators of correct folding

Recombinant expression was reported as arguably one of the most metabolically taxing activities that an organism could undergo [4]. It requires an abundance of resources in the form of energy and raw materials, and therefore there is a limit to the extent of resources each organism could allocate to such a task. When the resource demand surpassed an organism’s capacity, a stress response was observed, accompanied by a decrease in biomass production and growth rate due to the rewiring of metabolic fluxes in the cell [152]. Beyond energy and metabolite shortages, this stress response could also manifest itself in the form of cellular component shortages through changes in global gene expression [153].

Beyond folding patterns, the amino acid sequences of a protein can drastically impact the metabolic stress that *E. coli* may undergo during overexpression of exogenous proteins. The change of even a single amino acid residue was reported to impact the metabolic burden of *E. coli* during recombinant expression; these minor changes were shown to negatively impact cellular respiration activity and heterologous protein production levels [154]. Studies also revealed that silent exchanges in specific synonymous codons could impact growth, protein production levels, and respiration activity—demonstrating the growth sensitivity of *E. coli* to amino acid sequences [155]. Understanding codon biases and optimizing peptide sequences in accordance with the genetic makeup of the expression host was reported to be elemental in achieving a high-performing expression system [156].

Codon optimization was considered in only 16% of the work addressing issues around improving the recombinant protein expression performance of DtE enzymes in *E. coli*. It is possible that a conscious choice has been made not to codon optimize, as an expression strategy. The placement of rare codons with an mRNA region can promote stability in addition to ribosomal stalling allowing extra time for folding of problematic peptide regions [157]. These studies purchased synthetic genes from commercial manufacturers including GenScript USA Inc. [132, 158, 159], Invitrogen [96, 160], Sloning BioTechnology GmbH, and Synbio Technologies [47] that carry out codon optimisation on their products as a default service. The Graphical Codon Usage Analyser [41] and the Genescript Rare Codon Analysis Tool [72] were used for in-house codon analysis [6, 40]. This, however, does not rule out the possibility that codon optimization was carried out more extensively, but was not explicitly acknowledged in each respective publication. This ambiguity obscures the evidence about whether inclusion bodies were formed despite codon optimization or not and may limit the reproducibility of these experiments in the future. In select cases, DtE enzymes were expressed in commercial *E. coli* strains such as Rosetta, which were specifically recommended for alleviating codon bias in *E. coli* [27, 77–79] in addition to CodonPlus [67, 82, 161]; this may be a potential initial strategy to express a non-codon optimized gene.

### Cellular quality control mechanisms and the role of molecular chaperones

Molecular chaperones play an essential role in facilitating the recovery of misfolded protein aggregates. This in vivo quality control naturally exists within *E. coli* as its natural metabolism relies on cellular proteins that depend on appropriate folding patterns for proper function [162]. Recent research has found that chaperone systems such as GroEL/ES interact with a specific, smaller subset of the total proteome; this suggested that individual proteins in *E. coli*’s proteome were predetermined to be under the direct quality control management of a specific chaperone system such as GroEL/ES, Trigger factor (TF), or DnaKJE rather than this process being a random event [162, 163]. The challenge has been in determining which proteins would be assigned as substrates for specific molecular chaperones, and what attributes determine this distinction. Microarray studies showed that inclusion body formation led to the upregulation of genes associated with protein refolding and the heat shock genes, in addition to those associated with proteolysis [123]. Furthermore, molecular chaperones were reported to directly interact with aggregated recombinant protein products [164, 165].

Understanding the underlying factors that determine protein-chaperone interactions would be useful in ensuring that the correct chaperone would be favourably upregulated during protein expression. Chaperone-substrate interaction models were explored using metabolic network analysis techniques to understand the distribution of chaperone substrate enzymes in the metabolic network of *E. coli*; Takemoto et al. observed that metabolic enzymes that act as chaperone substrates became extensively distributed in the metabolic network as the chaperone requirements increased [163]. Although only limited amount of work has been reported in this field, detailed bioinformatics and metabolic network analyses could improve our understanding of the interaction patterns of molecular chaperones with recombinant proteins.

Analysis of sequence homology may prove useful to gain detailed insight into chaperone-substrate interaction patterns. Raineri et al. found closely related proteins from the *E. coli* and *Salmonella typhimurium* proteome, which were likely to show similar behavior and interact with the same or related chaperones in the GroEL system [166]. Therefore, sequence homology affects a recombinant enzyme’s interactions with molecular chaperones and has an indirect effect on the amount of product recovered from a misfolded state, and consequently on product titre. Mutations or changes introduced to the amino acid sequence of the peptide to be folded was reported to hinder the correct operation of the chaperone-mediated folding pathway [165]. This would be a risk to consider even in the case of beneficial mutations such as those introduced by site-directed mutagenesis to improve the catalytic activity of enzymes [158, 167–169].

Understanding these chaperone-substrate interaction patterns could be useful to selectively target and upregulate specific chaperone genes compatible with the DtE enzymes of interest to assist in folding. Co-expression of specific chaperones such as DnaK, DnaJ, GrpE, GroEL, GroES, or tig using commercial molecular chaperone plasmid sets from Takara Bio was observed in 12% of cases surveyed. This strategy proved useful in every case, with a varying degree of success depending on the study. However, this method is not as simple as expressing all chaperones at once. Soluble expression of *Psychrobacter* sp. lipase (Lip-498) 15 °C using pColdI plasmid was hindered by the individual co-expression of tig (pTf16) and GroEL/ES (pGro7) with the enzyme comprising 0.9% of total soluble proteins [170]. Lip-498 comprised 11.8% soluble protein when tig and GroEL/ES were co-expressed simultaneously. This value increased to 19.8% of total soluble protein when DnaK/DnaJ/GrpE and GroEL/ES (pG-KJE8) were co-expressed simultaneously. pGro7 and pG-KJE8 had the highest frequency of use among all commercial chaperone sets. Alternatively, a study by Peng et al. [103] showcased that native chaperones (Hsp60 and small heat-shock protein) of *Pyrococcus furiosus* can improve the soluble expression of its α-amylase in *E. coli* BL21 (DE3). It is unclear whether the co-expression of chaperones will always provide benefit, however, there is scope to investigate this hypothesis further.

### ‘Omics’-based investigation to improve experimental design and host genetic background

In the past, recombinant protein expression and its associated stress responses have been investigated at the ‘omic’-level in *E. coli* with the aim of improving heterologous expression performance. *E. coli* cells have demonstrated transcriptional changes at the global level in response inclusion bodies within the cytoplasm [123]. Genes taking an active role in protein folding (i.e. molecular chaperone genes), protein synthesis (i.e. aminoacyl-tRNA synthetases and ribosomal genes), and genes responsible for energy metabolism (e.g. ATP synthase) were observed to have a dynamic upregulation in response to the formation of inclusion bodies of recombinant protein fusions tagged with green fluorescent protein (GFP) [123]. Sharma et al. [171] provided a comparative analysis of how metabolic networks in *E. coli* BL21 (DE3) were reorganized in response to the physical state of the end protein product being soluble or being confined to inclusion bodies. Their study employed fed-batch cultures, mimicking industrial conditions, to overexpress rhIFN-b, xylanase and GFP; the transcription of amino acid biosynthesis and uptake genes was reported to be upregulated during inclusion body formation whereas the expression of these genes was downregulated during soluble expression, indicating that the solubility of the recombinantly expressed protein had a global impact on the transcription of the metabolic genes in *E. coli* [171].

The endometabolome of a cell is often thought to provide a physiological snapshot of a cell at a specific point in time. Chae et al. used two-dimensional NMR spectroscopy to evaluate the effect of stressors on the endometabolome of *E. coli*. They assessed the effects of elevated NaCl concentration as a stressor for *E. coli* expressing recombinant proteins; at high NaCl concentrations, the cells accumulated maltose and 2-hydroxy-3-methylbutanoic acid, which, in turn, promoted the solubility of two of the eleven aggregation prone proteins that were investigated in the study [172]. The names of these proteins were not explicitly mentioned in the text. Recombinant protein expression is a source of cellular stress on its own, therefore these fingerprinting studies can assist with pinpointing differences in the metabolite profiles of expression systems based on changes in the experimental conditions that the cells are exposed to during recombinant expression. The information gained at the metabolomic level could assist designing an experiment to redirect metabolic flux towards the intracellular accumulation of specific metabolites to overcome or alleviate inclusion body formation. Furthermore, inferences from this type of analysis can guide the choice of media. It was observed that factors including maintaining a pH 6 medium [45], the addition of betaine as an osmolyte [106], and the addition l-arginine [173] could improve the solubility of the final product in three studies, however ad hoc methods led to this discovery.

Supporting the design of expression studies with ‘omics’-based analyses could prove to be a useful strategy to improve solubility in addition to improving cellular biomass. The transcriptomic, metabolomic, and proteomic profiles of the *E. coli* expression hosts were under consideration in only one study within the scope of this review. This study discussed the use of metabolic engineering for the active production of xanthine dehydrogenase; their work demonstrated that the combinatorial overexpression of three global regulator and chaperone/helper proteins could improve the specific activity and solubility of their enzyme by up to 129% [174]. We proposed that ‘omic’-level information in combination with sequence-based modelling, codon optimization, or molecular chaperone studies could help us better understand *E. coli* as a production organism. Heterologous overexpression of proteins by *E. coli* can be considered to mimic the operation of a cell factory; ‘omics’-based technologies provide a level of process management over the operation by identifying the underlying bottlenecks in the manufacturing process in order to improve efficiency.

## Outlook: need for a systematic roadmap to address the demands of an expanding field

Our review of literature since 2010 showed that although the field of recombinant protein expression is associated with a plethora of knowledge, a systematic roadmap to help guide researchers to express problematic enzymes does not yet exist. We observe a variety of disparate practices and approaches adopted in the interest of promoting solubility, and the process is often led by ad hoc decisions. There is no standardized guideline for how enzyme expression is approached. Through experience researchers choose to adopt at least one method to preemptively reduce the possibility of their expression system to form inclusion bodies. Strategies could include inducing protein synthesis at low temperatures to reduce the rate of protein synthesis, and consequently promote correct folding, and to provide sufficient time for intracellular molecular chaperones to act [94, 156, 160]. This was a successful strategy in 14% of examples, with temperatures ranging from 10 to 25 °C as opposed to 37 °C, however even this strategy does not always lead to success solubility [175]. Reducing the inducer concentration, using plasmids with low copy numbers, or alternative promoters [176] are additional strategies employed.

The bulk of literature surveyed in this review focused on functional and structural characterization of enzymes. It appears that if the initial design led to soluble product, the follow-up experiments were conducted as planned. However, in the event that the heterologous protein product was not soluble, a trial-and-error approach was employed to achieve the correct combination of parameter settings to promote solubility. This approach was observed to be successful on individual cases but does not provide any guarantee of success. Each study employed quality control parameters in the form of catalytic activity assays to assess the final product; each quality control measure varied based on the specific enzyme evaluated. Expression system design may be dictated by previous experience or limited by the availability of the tools and materials in the individual lab in which the experiments were carried out. This may explain the limited use of specialized expression strains or alternative plasmid backbones, for example, that would provide an additional cost without a guarantee of success.

Our analysis has found no consensus in reporting basic aspects of the experimental protocol as indicated by these studies. The details on the gene of interest (i.e. its native host, amino acid sequence), how it was modified prior to expression (i.e. codon optimization carried out or not), and in certain cases the rationale behind how the vector was designed, or the choice of host, can all play an important role in shaping future work related to that expression system, as well as providing guidance for future studies. A lack of these details can lead to low reproducibility for research in this field. We believe that this necessitates the development of a minimum information standard scheme to systematize work in this field as a community effort, similar to existing efforts such as MIAPE or MIPFE for proteomics studies to standardize the reporting practices of experimental metadata relevant to structural and/or functional quality attributes of recombinant protein expression experiments [177, 178]. This information is required to facilitate reproducibility to build upon. A systematic collection of this standard metadata in database repositories in standalone format or accompanying any relevant experimental data is required. This metadata can also serve as foundation for modelling and bioinformatics in the field.

Amalgamated metadata detailing attempts to improve recombinant product solubility can potentially lead to the rapid discovery of broadly applicable rules for soluble enzyme expression in *E. coli*. While this review itself does not attempt to derive such rules, nor is there sufficient information available to derive these rules at this time, it is important to highlight this relevance. One effort that could assist the development of such a roadmap for recombinant enzymes would be to investigate the literature in which solubility of the enzyme product was successfully achieved from the start. Certain strategies used successfully for non-enzymatic protein including the use of inteins [179], reduced genome *E. coli* strains [180], or chromosomal integration [181, 182], though not discussed in this review, can provide further insights to support the efforts discussed above; however, the transferability of such techniques between protein types needs to be further understood. Furthermore, this volume of aggregated metadata highlighting all successful, partially successful, or unsuccessful experimental expression conditions can assist us to develop a strategic, evidence-based workflow for soluble recombinant enzyme production.

**Fig. 3:**
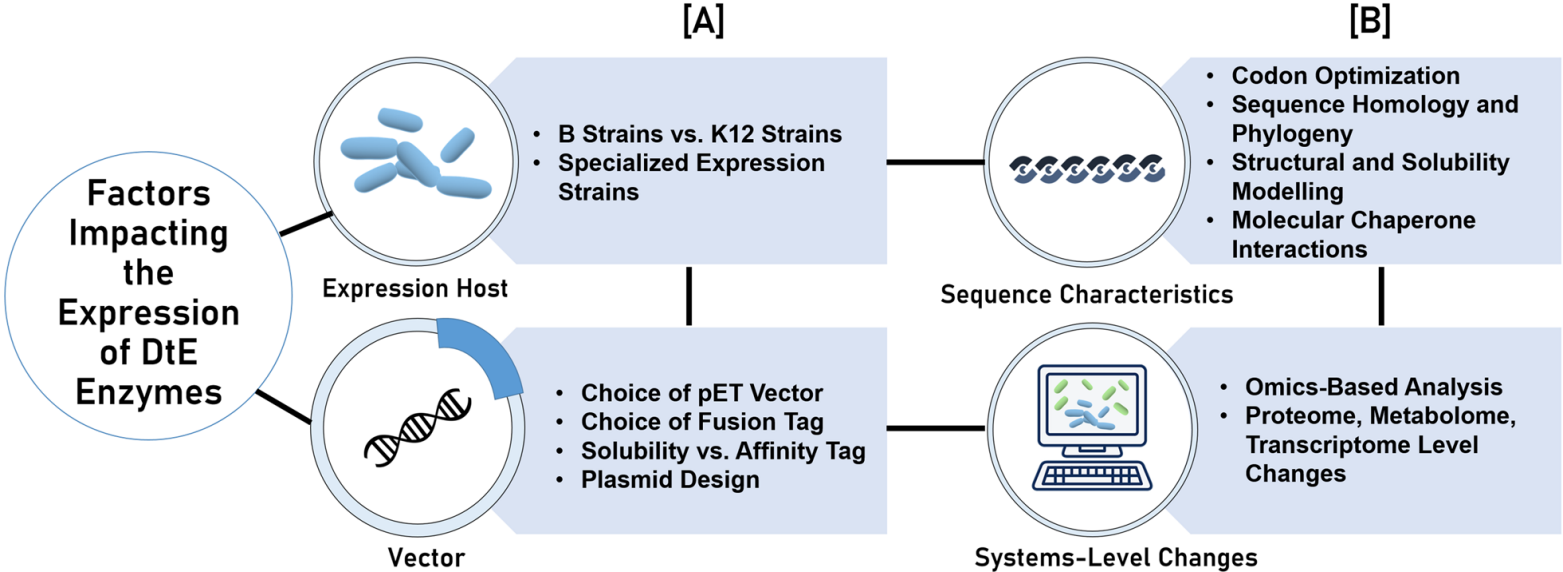
Summary of factors impacting the expression of difficult-to-express enzymes. **A** Parameters frequently explored and fine-tuned in experimental design, usually in an ad hoc manner; expression hosts and vector design. **B** Underlying factors that contribute to the formation of inclusion bodies and the bioinformatics and modelling tools used to evaluate the impact of these factors. The connecting lines demonstrate the interconnectivity of these parameters

The availability of a wide range of non-dominating options for the strains, vectors, and the design tools indicates a strong drive among researchers to have increased control over their experimental design to overcome the challenges that are associated with DtE enzymes. However, the current literature reveals a different landscape where these techniques were often underutilized or overlooked. This presents an opportunity to approach the challenge systematically. An understanding of the most frequently utilized tools—the expression strains, vectors, and the experimental conditions, can serve as a baseline for researchers to optimize their expression models from the start. In cases where this proves ineffective, the use of an integrated systems biology approach based on bioinformatics, modelling, and/or ‘omics’ technologies can highlight problematic pitfalls in the experimental design and provide additional information on the system of interest (Fig. 3). In other instances, these approaches can be adopted as a preliminary investigation before laboratory applications are made. The combination of all these approaches will assist in determining successful experimental conditions to recombinantly express a challenging candidate enzyme—promoting solubility.

## Supplementary Information

**Additional file 1: Table S1. **Experimental breakdown of all included publications.

## Data Availability

All data generated or analysed during this study are included in this published article (and its additional information files).

## Abbreviations

DtE: Difficult to express
SUMO: Small Ubiquitin-like Modifier
Trx: Thioredoxin
MBP: Maltose binding protein
His-tag: Polyhistidine-Tag
GST: Glutathione S-transferase
