# Supplementary material for: Recombinant expression of insoluble enzymes in Escherichia coli: a systematic review of experimental design and its manufacturing implications
Source: Microb Cell Fact. 2021 Oct 30;20:208. doi: 10.1186/s12934-021-01698-w (PMC8557517; doi:10.1186/s12934-021-01698-w)
Supplement: Supplementary file 1 — Additional file 1: Table S1. Experimental breakdown of all included publications. [file 12934_2021_1698_MOESM1_ESM.docx]

**Additional Information**

**Recombinant Expression of Insoluble Enzymes in *Escherichia coli* - A Systematic Review of Experimental Design and its Manufacturing Implications**

**Suraj Mital^1^, Graham Christie^1^, Duygu Dikicioglu^2^**

^1^Department of Chemical Engineering and Biotechnology, University of Cambridge,

Cambridge, United Kingdom CB3 0AS

^2^Department of Biochemical Engineering, University College London, London, United Kingdom WC1E 6BT

**Table S1**. Experimental breakdown of all included publications.

| **Publication Year** | **Expressed Enzyme** | **Enzyme Class** | **Industry of Use** | **Native Enzyme Source** | **Reason for Recombinant Expression** | ***E. coli* Expression Strain** | **Expression Vector** | **Fusion Tag** | **Author** |
| --- | --- | --- | --- | --- | --- | --- | --- | --- | --- |
| 2010 | Cellobiose Phosphorylase | transferase | waste/biomass processing | *Cellulomonas gilvus* | Not mentioned | JM109 | pUC18 | No Mention | [1] |
| 2010 | S-adenosyl-L-methionine (SAM)–dependent methyltransferase | oxidoreductase | pharmaceuticals | *Escherichia coli* K-12 Strain | Not mentioned | K-12 strain MG1655 | pET28b+ | His-tag | [2] |
| 2010 | Lysine 6-dehydrogenase | oxidoreductase | chemical | *Geobacillus stearothermophilus* | Difficult to culture organism in lab - thermophile | Rosettagami (DE3) | pColdI | No Mention | [3] |
| 2010 | glycosyl transferase | isomerase | biocatalysis | *Streptomyces* sp. 139 | Not mentioned | DH5α | pUC18 | His-tag | [4] |
| 2010 | Cysteine desulfurase | transferase | chemical | *Anabaena* sp. strain PCC7120 | Not mentioned | BL21(DE3) | pET22 | His-tag | [5] |
| 2010 | Aminopentol aminotransferase | transferase | pharmaceuticals, agriculture | *Sphingopyxis*sp. MTA144 | Difficulty expressing in other hosts such as *pichia pastoris* | 1. BL21(DE3) 2. HMS174(DE3) 3. Origami 2 (DE3)  4. Rosetta 2 (DE3) 5. Rosetta-gami (DE3) 6. Tuner (DE3)  7.ArcticExpress (DE3) | pET3a, pET30a, pMAL | His-tag, MBP | [6] |
| 2010 | Alpha-cyclodextrin glucanotransferase | Transferase | starch processing | *Paenibacillus macerans* | Not mentioned | BL21 (DE3) | pET20b(+) | No Mention | [7] |
| 2010 | histidine kinase | transferase | pharmaceutical | *﻿Brucella abortus* | Not mentioned | MG1655, Top10 | pBluescriptKS | mcherry for reporting | [8] |
| 2010 | ﻿cholesterol oxidase | oxidoreductase | pharmaceutical, agriculture | *﻿Brevibacterium sterolicum* | Not mentioned | ﻿BL21(DE3)pLysS | pET24b | His-tag (c-terminal) | [9] |
| 2010 | ﻿curdlan synthase | transferase | food | *﻿Agrobacterium* sp. | Not mentioned | 1. Origami (DE3), 2. Origami B (DE3) 3. BL21 (DE3) 4. Rosetta-Gami | pET32a, ﻿Champion™ pET SUMO | His-tag (N-terminal), SUMO | [10] |
| 2010 | ﻿phosphorylcholine phosphatase | hydrolase | pharmaceutical | *﻿Pseudomonas aeruginosa* | Safety concerns - infectious organism | ﻿BL21(DE3) | pET9b | no mention | [11] |
| 2010 | ﻿tetrathionate hydrolase | hydrolase | chemical | *﻿Acidithiobacillus ferrooxidans* ﻿ATCC23270 | Not mentioned | BL21 Star (DE3) | pET4 | no mention | [12] |
| 2011 | Lipase | hydrolase | food, detergent, pharmaceutical | *Serratia marcescens* | lower expression | BL21(DE3) | pET24a(+) | no-tag | [13] |
| 2011 | chitobiase | hydrolase | Agriculture (seafood) | *Escherichia coli* K-12 Strain | Not mentioned | M15 | pQE30 | His-tag | [14] |
| 2011 | p-coumaric acid decarboxylase | oxidoreductase | food | *Lactobacillus plantarum* | Difficult purification strategies | JM109 (DE3) | pURI | His-tag | [15] |
| 2011 | lumazine synthase | transferase | pharmaceuticals | *Brucella* sp. | Not mentioned | BL21(DE3) | pET11a | No Mention | [16] |
| 2011 | Tryptophan indole-lyase | lyase | chemical | *Photobacterium profundum* (strain SS9) | Not mentioned | BL21(DE3) | pET100 | no-tag | [17] |
| 2011 | Lipase | hydrolase | food, biocatalysis | *Geobacillus* sp. SBS-4S | Difficult to culture organism in lab - thermophile | BL21(DE3) | pET21a | No Mention | [18] |
| 2011 | Quinoprotein glucose dehydrogenase B | oxidoreductase | biosensors/diagnostics | *Acinetobacter calcoaceticus* | Not mentioned | NovaBlue (DE3) | pRSF1b | His-tag (c-terminus) | [19] |
| 2011 | Beta-lactamase | hydrolase | pharmaceuticals | *Zymomonas mobilis* ZM4 | Not mentioned | BL21(DE3) | pZTE103 | No Mention | [20] |
| 2011 | arylamine N-acetyltransferase | transferase | pharmaceuticals | *Mycobacterium tuberculosis* H37Rv | Not mentioned | BL21(DE3)pLysS | pET28b(+) | His-tag (n-terminal) | [21] |
| 2011 | ﻿polyhydroxyoctanoate depolymerase | hydrolase | chemical, bioplastics | *﻿Pseudomonas putida* KT2442 | Not mentioned | ﻿M15 | ﻿pREP4, pPAZ3 | His-tag | [22] |
| 2011 | ﻿lipase B | hydrolase | detergent | *﻿Candida antarctica* | Not mentioned | ﻿Origami 2TM (DE3) | ﻿pColdIII | ﻿polycationic tags (10 Arginine residues, 10 Lysine residues, 10 histidine residues) at either C or N terminus | [23] |
| 2011 | ﻿lipase Lip-948 | hydrolase | chemical, detergent | *﻿Psychrobacter* sp. G | Difficult to culture organism in lab - thermophile (cold) | ﻿BL21(DE3) | ﻿pColdI | His-tag | [24] |
| 2012 | Fuculose-1-Phosphate Aldolase | lyase | chemical, pharmaceutical | *Escherichia coli* K-12 Strain | Not mentioned | XL1 Blue and JGT20 | pTrcfuc | His-tag (n-terminal) | [25] |
| 2012 | cyclohexanone monooxygenase | oxidoreductase | pharmaceuticals | *Polaromonas* sp. (strain JS666) | Not mentioned | BL21 Star (DE3) | pET 101/D-TOPO | No Mention | [26] |
| 2012 | exo-oligoalginate lyase | lyase | biofuels | *Saccharophagus degradans* | Not mentioned | BL21(DE3) | pET21a | His-tag | [27] |
| 2012 | NAD-specific glutamate dehydrogenase | oxidoreductase | biosensors/diagnostics | *Halobacterium salinarum* | Difficult to culture organism in lab - halophile | BL21(DE3) | pRV1-tna | No Mention | [28] |
| 2012 | Styrene monooxygenase | oxidoreductase | pharmaceutical, agrochemical | *Rhodococcus* sp. ST-5 | Not mentioned | BL21(DE3) | pET21b(+) | His-tag (c-terminus) | [29] |
| 2012 | Flavin oxidoreductase | oxidoreductase | pharmaceutical, agrochemical | *Rhodococcus* sp. ST-10 | Not mentioned | BL21(DE3) | pET21b(+) | His-tag (c-terminus) | [29] |
| 2012 | lipase | hydrolase | detergent, food, paper | *Marinobacter lipolyticus* | Difficult to culture organism in lab - halophile | BL21(DE3) | pET28b+ | His-tag (c-terminus) | [30] |
| 2012 | Hydroquinone dioxygenase large subunit | oxidoreductase | chemical, plastics, dyes | *Burkholderia* sp. SJ98 | Not mentioned | BL21 AI | pDest | His-tag (n-terminal) | [31] |
| 2012 | ﻿asparaginase II | hydrolase | food, pharmaceutical | *Escherichia coli* K-12 strain (JM109) | Not mentioned | ﻿ BL21 (DE3) | ﻿pET14b | His-tag (N-terminal) | [32] |
| 2012 | ﻿glutamate-specific endopeptidase | hydrolase | pharmaceutical | *﻿Bacillus licheniformis* | Not mentioned | ﻿BL21(DE3) | pET22b | His-tag (c-terminal) | [33] |
| 2012 | ﻿DNA gyrase | isomerase | pharmaceutical | *﻿Chlamydia trachomatis* | Not mentioned | ﻿BL21(DE3) | pET100, pET101 | His-tag | [34] |
| 2012 | ﻿flavin reductase | oxidoreductase | chemical, pharmaceutical | ﻿*Sulfolobus tokodaii* strain 7 | Difficult to culture organism in lab - thermoacidophile | ﻿Rosetta (DE3) | pET28a | His-tag (N-terminal) | [35] |
| 2012 | ﻿alginate lyase | lyase | food, pharmaceutical | *﻿Pseudomonas alginovora* | Not mentioned | ﻿BL21(DE3), C41(DE3) and C43(DE3) | pFO4 (peT15a derivative), pGEX-4t-1 | His-tag, GST tag (n-terminal) | [36] |
| 2012 | ﻿glucose-1-phosphate thymidylyltransferase | transferase | pharmaceutical | *﻿Streptomyces peucetius* | Not mentioned | ﻿BL21(DE3) | pET32a | His-tag (N-terminal) | [37] |
| 2012 | ﻿diguanylate cyclase | transferase | pharmaceutical | *﻿Thermotoga maritima* | Not mentioned | ﻿BL21(DE3) | pET24b | His-tag (c-terminal) | [38] |
| 2013 | proline dehydrogenase | oxidoreductase | pharmaceutical, biosensors | *Pseudomonas fluorescens* | expensive, complicated purification methods from native host | BL21(DE3) | pET23a | No Mention | [39] |
| 2013 | Lipase | hydrolase | food, biocatalysis | *Thermomyces lanuginosus* | Not mentioned | BL21(DE3) | pJExpress401 | His-tag | [40] |
| 2013 | Flavin reductase | oxidoreductase | water treatment | *Citrobacter freundii* | Difficult purification methods for this enzyme | BL21(DE3) pLysS | pET43.1c(+) | No Mention | [41] |
| 2013 | Dibenzothiophene sulfone monoxygenase | oxidoreductase | fossil fuel treatment | *Gordonia alkanivorans* | Not mentioned | BL21 | pET21a | His-tag (n-terminal) | [42] |
| 2013 | 2-hydroxybiphenyl-2-sulfinate sulfinolyase | hydrolase | fossil fuel treatment | *Gordonia alkanivorans* | Not mentioned | BL21 | pET21a | His-tag (n-terminal) | [42] |
| 2013 | Alpha-amylase | hydrolase | starch processing | *Exiguobacterium* sp. DAU5 | Difficult to culture organism in lab - thermophile | BL21 | pET32a (+) | His-tag (n-terminal) | [43] |
| 2013 | Bis-γ-glutamylcystine Reductase | oxidoreductase | chemical, remediation | *Halobacterium salinarum* | Not mentioned | ArcticExpress (DE3) | pET46 | His-tag (n-terminal) | [44] |
| 2013 | Tk-subtilisin | hydrolase | food, detergent | *Thermococcus kodakarensis* | Not mentioned | BL21(DE3) | pET25b | No Mention | [45] |
| 2013 | Laccase | oxidoreductase | chemical processing | *Bacillus* sp. HR03 | Very low expression | BL21 (DE3) | pET21a (+) | No Mention | [46] |
| 2013 | ﻿hybrid cellulase | hydrolase | textile, paper | ﻿*Sulfolobus solfataricus* P2 | Not mentioned | strain BL21 AI | pET28C | No Mention | [47] |
| 2013 | L-proline 4-hydroxylase | oxidoreductase | biocatalysis | *Dactylosporangium* sp. | Not mentioned | ﻿BL21(DE3) | pET24a | no mention | [48] |
| 2013 | ﻿arginine deiminase | hydrolase | pharmaceutical | *﻿Mycoplasma hominis* | Not mentioned | ﻿BL21(DE3) | pET3d | no mention | [49] |
| 2013 | ﻿uridine phosphorylase | transferase | biocatalysis | *﻿Aeropyrum pernix* K1 | Not mentioned | ﻿BL21(DE3) | pET28a, pET30a | His-tag | [50] |
| 2013 | l-amino acid deaminase | oxidoreductase | chemical | *﻿Proteus mirabilis* | Not mentioned | ﻿ BLL21(DE3) | pET20b | His-tag | [51] |
| 2013 | ﻿a-amylase | hydrolase | starch processing | *﻿Bacillus aquimaris* MKSC 6.2 | Not mentioned | ﻿BL21(DE3) | pET30 | No Mention | [52] |
| 2013 | ﻿acid phosphatase | hydrolase | food | *﻿Enterobacter aerogenes* IAM1183 | Not mentioned | BL21(DE3) | pET28a | His-tag | [53] |
| 2013 | minor extracellular (Epr) protease | hydrolase | food | *﻿Bacillus licheniformis* strain USC13 | Not mentioned | ﻿BL21(DE3) | pET30a | no mention | [54] |
| 2013 | ﻿Ferrochelatase | lyase | pharmaceutical, medicine | *﻿Synechocystis* sp. PCC 6803 | Not mentioned | Rosetta 2 (DE3) | pET15b | His-tag (N-terminal) | [55] |
| 2013 | ﻿lipase | hydrolase | pharmaceutical, food, chemical | *﻿Psychrobacter cryohalolentis* K5T | ﻿Difficult to culture psychotropic host | ﻿BL21(DE3) | pET32a | His-tag (c-terminal) | [56] |
| 2014 | pullulanase | hydrolase | food | *Thermus thermophilus* | Difficult to culture organism in lab - thermophile and halophile | BL21(DE3) | pET28a (+) | His-tag (c-terminus) | [57] |
| 2014 | Lipase | hydrolase | Food | *Bacillus amyloliquefaciens* | Not mentioned | BL21(DE3) | pET28a (+) | His-tag | [58] |
| 2014 | ssfS3 | oxidoreductase | pharmaceuticals | *Streptomyces* sp. SF2575 | Not mentioned | BL21(DE3) | pKW423 | His-tag | [59] |
| 2014 | SsfS4 | oxidoreductase | pharmaceuticals | *Streptomyces* sp. SF2575 | Not mentioned | BL21(DE3) | pKW423 | His-tag | [59] |
| 2014 | SsfS5 | oxidoreductase | pharmaceuticals | *Streptomyces* sp. SF2575 | Not mentioned | BL21(DE3) | pKW423 | His-tag | [59] |
| 2014 | SsfS6 | oxidoreductase | pharmaceuticals | *Streptomyces* sp. SF2576 | Not mentioned | BL21(DE3) | pKW423 | His-tag | [59] |
| 2014 | Lipase | hydrolase | food, biocatalysis | *Mycobacterium tuberculosis* | Not mentioned | M15 | pQE30UA | His-tag | [60] |
| 2014 | NADH:flavin oxidoreductase | oxidoreductase | biocatalysis | *Acinetobacter baylyi* | Not mentioned | BL21 (DE3) | pJET1.2 | No Mention | [61] |
| 2014 | 2-hydroxyethylphosphonate methyltransferase | transferase | chemical, agrichemical | *Streptomyces wedmorensis* | Not mentioned | Rosetta 2 (DE3) pLysS | pET30a(+) | His-tag (n-terminal) | [62] |
| 2014 | Phenol hydroxylase component 2 | oxidoreductase | chemical | *Rhodococcus opacus* | Not mentioned | BL21-CodonPlus-RP | pET11a | No Mention | [63] |
| 2014 | pullulanase | hydrolase | starch processing | *Bacillus acidopullulyticus* | Not mentioned | BL21 (DE3) | pET20b(+) | His-tag, GST (n-terminal) | [64] |
| 2014 | ﻿Poly(vinyl alcohol) dehydrogenase | oxidoreductase | textile, paper, chemical | *﻿Sphingopyxis* sp. 113P3 | Not mentioned | ﻿Rosetta (DE3) | ﻿pET32a | TRX tag (n-terminal) | [65] |
| 2014 | arignine deaminase | hydrolase | pharmaceutical | *﻿Pseudomonas putida* | Not mentioned | ﻿BL21(DE3) | ﻿pET30a(+), pBAD/gIIIA, pColdTF and pGEX-6P-1 | His-tag on pET30 plasmid | [66] |
| 2014 | ﻿Streptokinase | hydrolase | pharmaceutical | *﻿Streptococcus pyogenes* DT7 | Lower yield | ﻿BL21(DE3) | pET22b | His-tag (c-terminal) | [67] |
| 2014 | ﻿nitrile hydratase | lyase | biocatalysis, bioremediation | *﻿Pseudomonas putida* | Not mentioned | ﻿BL21(DE3) | pET24a | no mention | [68] |
| 2014 | ﻿b-galactosidase | hydrolase | food | *﻿Pyrococcus furiosus* | Not mentioned | ﻿BL21(DE3) | pET24a | No Mention | [69] |
| 2014 | ﻿glucoamylase | hydrolase | starch processing | *﻿Thermoanaerobacter tengcongensis* | Not mentioned | ﻿Rosetta (DE3) | pET21a, pET28a | His-tag (c-terminal) | [70] |
| 2015 | 6‐phosphofructokinase | transferase | food | *Thermus thermophilus* HB8 | Difficult to culture organism in lab - thermophile | DH5α | pUC19 | No Mention | [71] |
| 2015 | Fructose-1,6-bisphosphate aldolase | lyase | food | *Thermus thermophilus*HB9 | Difficult to culture organism in lab - thermophile | Dh5α | pUC20 | No Mention | [71] |
| 2015 | Transglutaminase | transferase | chemical, pharmaceutical | *Streptomyces mobaraensis* | Toxic at high expression levels | JM109 | pUC18 | No Mention | [72] |
| 2015 | Type I pullulanase | hydrolase | food, waste management, textile | *Shewanella arctica* | Difficult to culture organism in lab - thermophile (cold) | M15 | pQE-30 | His-tag (n-terminal) | [73] |
| 2015 | beta-glucosidase (gh2) | hydrolase | food, biofuels | *Thermus brockianus* | Difficult to culture organism in lab - thermophile | BL21 StarTM(DE3) | pQE80L | No Mention | [74] |
| 2015 | lysine-ε-oxidase | oxidoreductase | chemical | *Marinomonas mediterranea* | Not mentioned | Rosetta | pET15 | His-tag (n-terminal) | [75] |
| 2015 | Carboxypeptidase T | hydrolase | food | *Thermoactinomyces vulgaris* | Not mentioned | BL21(DE3)pLysS | pET15b | No Mention | [76] |
| 2015 | ﻿Pseudolysin | hydrolase | pharmaceutical | *﻿Pseudomonas aeruginosa* | Lower yield | ﻿BL21(DE3) | pET28a | His-tag | [77] |
| 2015 | ﻿nitrile hydratase | lyase | biocatalysis, bioremediation | *﻿Aurantimonas manganoxydans* | Not mentioned | ﻿BL21(DE3) | ﻿pET28a (+) and pCDFDuet-1 | His-tag (N-terminal) | [78] |
| 2015 | ﻿Pectate lyase | lyase | food, paper | *﻿Bacillus subtilis* RCK | Previously used secretion strain of *subtilis* | ﻿ BL21(DE3) | pET28a | no mention | [79] |
| 2015 | ﻿N-Acyl-D-glucosamine 2-epimerase | isomerase | pharmaceutical | *﻿Anabaena variabilis* ATCC 29413 | Not mentioned | 1. BL21(DE3) 2.Tuner (DE3)  3.C41(DE3)  4.C43(DE3) | pET28a | The use of multiple fusion tags MBP, His-tag, SBP, GST, SUMO | [80] |
| 2015 | ﻿Cholesterol oxidase | oxidoreductase | pharmaceutical, agriculture | *﻿Pseudomonas aeruginosa* | Not mentioned | ﻿Rosetta | pET21a | no mention | [81] |
| 2015 | chitinase | hydrolase | pharmaceutical, agriculture, bioremediation | *﻿Halobacterium salinarum* | Not mentioned | 1. BL21 (DE3)  2.BL21 Star (DE3)  3. Tuner (DE3), Rosetta 2 (DE3)  4. BL21 RP (DE3) | ﻿pET100/D-TOPO, ﻿pETite | His-tag | [82] |
| 2015 | Lipase | hydrolase | chemical, detergent | *﻿Pseudomonas moraviensis* M9 | Not mentioned | ﻿BL21(DE3) | pET22b | His-tag | [83] |
| 2016 | ﻿serine protease | hydrolase | chemical | *﻿Thermus thermophilus* HB8 | Not mentioned | ﻿Transetta (DE3) | pET22b | His-tag (c-terminal) | [84] |
| 2016 | β-agarase | hydrolase | food, cosmetics | *Flammeovirga*sp. SJP92 | Not mentioned | BL21(DE3) | pET28m | SUMO | [85] |
| 2016 | tyrosinase | oxidoreductase | food, bioremediation | *Nitrosopumilus koreensis* | Not mentioned | BL21(DE3) | pET23b+ | His-tag | [86] |
| 2016 | Putative Cellulase | hydrolase | textile, food processing | uncultured bacterium (metagenomic study) | Not mentioned | Rosetta (DE3) | pET15b | His-tag (n-terminal) | [87] |
| 2016 | Tryptophan 2-C-methyltransferase | transferase | pharmaceuticals | *Streptomyces laurentii* | Not mentioned | RosettaBlue (DE3) pLysS | pSUMO | His-tag (n-terminal) | [88] |
| 2016 | Sphingomyelinase | hydrolase | pharmaceuticals | *Mycobacterium tuberculosis* ATCC 25618 | Not mentioned | Rosetta 2 (DE3) | pET22b | His-tag | [89] |
| 2016 | Neopentalenolactone D synthase | oxidoreductase | chemical | *Streptomyces avermitilis* | Not mentioned | BL21(DE3) | pET28a | His-tag (n-terminal) | [90] |
| 2016 | Nattokinase | hydrolase | Pharmaceuticals | *Bacillus subtilis* var. natto | Low yield and difficulty in purification  Expression in P. pastoris and L. lactis was also very low | BL21 (DE3) | pQE30 | No Mention | [91] |
| 2016 | Sarcosine oxidase | oxidoreductase | pharmaceuticals | *Thermomicrobium roseum* | Difficult culturing conditions of native host - thermophile | BL21 (DE3) | pET28a (+) | No Mention | [92] |
| 2016 | Lysyl endopeptidase | hydrolase | pharmaceuticals | *Pseudomonas aeruginosa* | Very low expression | BL21 (DE3) | pET32a (+) | TRX-Tag | [93] |
| 2016 | ﻿alcohol dehydrogenase | oxidoreductase | biocatalysis, chemical | *﻿Rhodococcus erythropolis* | Not mentioned | ﻿BL21(DE3) | ﻿pACYC-MEP | His-tag | [94] |
| 2016 | Chitobiase | hydrolase | Agriculture | Escherichia coli (strain K12) | Not mentioned | M15 | pQE-30 | No Mention | [95] |
| 2016 | ﻿AMS8 lipase | hydrolase | detergent, food | *﻿Pseudomonas* sp. | Difficult to culture organism in lab - thermophile (cold) | ﻿BL21(DE3) | pET32b | His-tag (N-terminal) | [96] |
| 2016 | ﻿α-amylase | hydrolase | starch processing | *﻿Pyrococcus furiosus* | Extracellular protein difficult to express in large quantity | ﻿BL21(DE3) | ﻿pACYCDuet-1, pCDFDuet-1 | no mention | [97] |
| 2017 | Xanthine dehydrogenase | oxidoreductase | pharmaceutical | *Rhodobacter capsulatus* | Complicated extraction protocol, originally taken from milk or low expression in microbes | BL21(DE3) | pTrc99A | No Mention | [98] |
| 2017 | α-Amylase | hydrolase | baking, brewing, distilling, textiles, detergents, and paper | *Arthrobacter agilis* | Difficult to culture organism in lab - thermophile | BL21(DE3) | pET28a | His-tag (n-terminal) | [99] |
| 2017 | α-amylase | hydrolase | starch processing | *Thermococcus* sp. HJ21 | Difficult to culture organism in lab - thermophile | BL21(DE3) | pET28b | His-tag (n-terminal) | [100] |
| 2017 | Carbendazim hydrolase | hydrolase | remediation | *Mycobacterium* sp. | Not mentioned | BL21(DE3) | pET29a | No Mention | [101] |
| 2017 | ﻿fructotranslyaferase | transferase | biocatalysis | *﻿Aspergillus oryzae* | Lower yield | BL21(DE3) | pET32a | His-tag | [102] |
| 2017 | ﻿3-ketosteroid-Δ1-dehydrogenase | oxidoreductase | chemical | *﻿Mycobacterium neoaurum* | Not mentioned | ﻿BL21(DE3) | pET28a | no mention | [103] |
| 2018 | Chondroitin sulfate ABC endolyase I | lyase | chemical, pharmaceutical | *Proteus vulgaris* | Lower expression and activity levels | BL21(DE3) | pET15D | His-tag (n-terminal) | [104] |
| 2018 | Phospholipase A1 | hydrolase | food processing | *Yersinia pseudotuberculosis* | Membrane protein, so produced in lower levels | BL21(DE3) | pET32 | No Mention | [105] |
| 2018 | Phosphoenolpyruvate carboxylase | Lyase | food | *Leuconostoc mesenteroides* ATCC 8293 | Not mentioned | BL21(DE3) | pET28a | His-tag | [106] |
| 2018 | Malolactic enzyme | lyase | food | *Leuconostoc mesenteroides* ATCC 8293 | Not mentioned | BL21(DE3) | pET28a | His-tag | [106] |
| 2018 | Sialidase (nanH1) | hydrolase | pharmaceuticals | *Bacteroides fragilis* | Native host produces a glycosylated version of this enzyme, the researchers wanted a non-glycosylated version | BL21 (DE3) | pET28a (+) | His-tag | [107] |
| 2018 | l-Asparaginase | hydrolase | pharmaceutical, food | *Aspergillus terreus* | Not mentioned | ﻿Bl21 (DE3) pLysS | pET28a | His-tag | [108] |
| 2019 | 2-aminoethylphosphonate-pyruvate transaminase | transferase | pharmaceuticals | *Bradyrhizobium elkanii* | Not mentioned | BL21(DE3) | pET30a | no-tag | [109] |
| 2019 | Formate dehydrogenase | oxidoreductase | biocatalysis | *Cupriavidus necator* | Not mentioned | BL21 (DE3) | pET17b | No Mention | [110] |
| 2019 | Aryl-alcohol oxidase | oxidoreductase | chemical | *Pleurotus eryngii* | Low yield from native host, making production of enzyme expensive | W3110 | pFLAG1 | No Mention | [111] |
| 2019 | Pyruvate oxidase | oxidoreductase | Biotechnology (biosensors and bioanalytics) | *Aerococcus viridans* | Low expression yields in native host | BL21 (DE3) | pET28a-pod | No Mention | [112] |
| 2019 | Malto-oligosyltrehalose trehalohydrolase | hydrolase | starch processing | *Sulfolobus acidocaldarius* | Not mentioned | BL21 (DE3), Origami (DE3) | pET-24a (+), pET-32a (+) | TRX-Tag | [113] |
| 2019 | ﻿β-galactosidase | hydrolase | food | *Escherichia coli* K-12 (W3110) | Not mentioned | BL21λ Codon plus | ﻿pET 26+ | His-tag (c-terminal) | [114] |
| 2019 | ﻿serratiopeptidase | hydrolase | detergent, food, paper | *﻿Serratia marcescens* | Low yield from native host | BL21(DE3) 2. C43(DE3)  3. BL21(DE3)-pLysS 4.Rosetta(DE3)-pLysS | ﻿pET23b(+) | No Mention | [115] |
| 2019 | ﻿trehalose transferase | Transferase | food, pharmaceutical | *﻿Pyrobaculum yellowstonensis* | Not mentioned | ﻿Top10 | ﻿pBAD/His A | His-tag (both C and N terminal) | [116] |
| 2019 | ﻿manganese catalase | oxidoreductase | ﻿food, medical, paper, textile | *﻿Geobacillus thermopakistaniensis* | Not mentioned | ﻿BL21-CodonPlus (DE3)-RIL cells | pET21a | His-tag | [117] |
| 2019 | ﻿serine protease | hydrolase | chemical | *﻿Thermus thermophilus* HB8 | Not mentioned | ﻿Transetta (DE3) | pET22b | His-tag (c-terminal) | [118] |
| 2019 | laccase | oxidoreductase | bioremediation | *Yersinia enterocolitica* strain 8081 | Not mentioned | ﻿BL21(DE3) | pET28a | His-tag | [119] |
| 2019 | lipase | hydrolase | biofuel | *Proteus* sp. NH 2-2 | Not mentioned | ﻿BL21(DE3) | pET28a | His-tag | [120] |
| 2019 | ﻿prenyltransferase | transferase | pharmaceutical | *﻿Streptomyces niveus* | Not mentioned | 1. BL21 (DE3)  2. Rosetta™ (DE3)  3.ArcticExpress (DE3) RP cells | pET28a, pET32a, pGEX4T-1 and pETDuet-1 | His-tag, TRX, GST, MBP | [121] |
| 2019 | ﻿polyhydroxyalkanoate synthase | transferase | bioplastic | *﻿Ralstonia eutropha* | Not mentioned | ﻿BL21(DE3) | pGEM | GST tag | [122] |
| 2019 | ﻿multicopper oxidase | oxidoreductase | pharmaceutical | *﻿Mycobacterium tuberculosis* | Not mentioned | ﻿BL21(DE3) | pET21a | His-tag | [123] |
| 2019 | laccase | oxidoreductase | textile | *Bacillus subtilis* strain R5 | Not mentioned | ﻿BL21-CodonPlus(DE3)-RIL | pET28a | no mention | [124] |
| 2020 | D-Galactose-specific L-arabinose isomerase | oxidoreductase | food | *Bifidobacterium adolescentis* | Not mentioned | BL21(DE3) | pANY1 | No Mention | [125] |
| 2020 | Glucose Dehydrogenase | oxidoreductase | chemical | *Oscillatoria* sp. PCC 10802 | Not mentioned | BL21 (DE3) | pET21a(+) | No Mention | [126] |
| 2020 | Sucrose Phosphorylase | transferase | Food, cosmetic, pharmaceutical | *Thermoanaerobacterium thermosaccharolyticum* | Poor thermostability and low expression levels limit | BL21 (DE3) | pET20b (+) | No Mention | [127] |
| 2020 | ﻿creatinine deiminase | hydrolase | chemical, pharmaceutical | *﻿Corynebacterium glutamicum* | Not mentioned | ﻿BL21(DE3) | pET32a | His-tag (N-terminal) | [128] |
| 2020 | Laccase | oxidoreductase | ﻿textile, paper, bioremediation, chemical, biosensing, biofuel | *﻿Geobacillus* sp. JS12 | Not mentioned | ﻿BL21(DE3) | pET21a | His-tag | [129] |
| 2020 | ﻿nuclease | hydrolase | pharmaceutical, food | *﻿Yersinia enterocolitica* subsp. palearctica | Not mentioned | ﻿ Rosetta 2(DE3) | pET24a | no mention | [130] |
| 2020 | ﻿lipase | hydrolase | ﻿chemical, food, detergent, pulp and paper | *﻿Pseudomonas fluorescence* KE38 | Not mentioned | ﻿BL21(DE3) | pET28a | His-tag (N-terminal) | [131] |
| 2021 | ﻿carbonic anhydrase | lyase | CO2 sequestering, biofuel | *﻿Mesorhizobium loti* | Not mentioned | BL21(DE3) | pET28a, pET32a | TRX tag | [132] |
| 2021 | ﻿cellulase | hydrolase | textile, detergent, paper | *﻿Bacillus subtilis* (natto CGMCC2108 strain) | Not mentioned | ﻿BL21(DE3) | pET21a | His-tag | [133] |

**Additional File 1.** List of publications included in systematic review and meta-analysis

1. Singh SP, Purohit MK, Aoyagi C, Kitaoka M, Hayashi K. Effect of growth temperature, induction, and molecular chaperones on the solubilization of over-expressed cellobiose phosphorylase from Cellvibrio Gilvus under in vivo conditions. Biotechnology and Bioprocess Engineering [Internet]. 2010;15:273–6. Available from: http://link.springer.com/10.1007/s12257-009-0023-1

2. Lin S, Hanson RE, Cronan JE. Biotin synthesis begins by hijacking the fatty acid synthetic pathway. Nature Chemical Biology [Internet]. Nature Publishing Group; 2010;6:682–8. Available from: http://www.nature.com/articles/nchembio.420

3. Yoneda K, Fukuda J, Sakuraba H, Ohshima T. First Crystal Structure of l-Lysine 6-Dehydrogenase as an NAD-dependent Amine Dehydrogenase. Journal of Biological Chemistry [Internet]. 2010;285:8444–53. Available from: http://www.jbc.org/lookup/doi/10.1074/jbc.M109.084384

4. Li X, Wang L, Bai L, Yao C, Zhang Y, Zhang R, et al. Cloning and characterization of a glucosyltransferase and a rhamnosyltransferase from Streptomyces sp. 139. Journal of Applied Microbiology [Internet]. 2010;108:1544–51. Available from: http://doi.wiley.com/10.1111/j.1365-2672.2009.04550.x

5. Ruiz M, Bettache A, Janicki A, Vinella D, Zhang C-C, Latifi A. The alr2505 (osiS) gene from Anabaena sp. strain PCC7120 encodes a cysteine desulfurase induced by oxidative stress. FEBS Journal [Internet]. 2010;277:3715–25. Available from: http://doi.wiley.com/10.1111/j.1742-4658.2010.07772.x

6. Hartinger D, Heinl S, Schwartz H, Grabherr R, Schatzmayr G, Haltrich D, et al. Enhancement of solubility in Escherichia coli and purification of an aminotransferase from Sphingopyxis sp. MTA144 for deamination of hydrolyzed fumonisin B1. Microbial Cell Factories [Internet]. 2010;9:62. Available from: http://microbialcellfactories.biomedcentral.com/articles/10.1186/1475-2859-9-62

7. Li Z, Gu Z, Wang M, Du G, Wu J, Chen J. Delayed supplementation of glycine enhances extracellular secretion of the recombinant α-cyclodextrin glycosyltransferase in Escherichia coli. Applied Microbiology and Biotechnology [Internet]. 2010;85:553–61. Available from: http://link.springer.com/10.1007/s00253-009-2157-7

8. van der Henst C, Charlier C, Deghelt M, Wouters J, Matroule JY, Letesson JJ, et al. Overproduced Brucella abortus PdhS-mCherry forms soluble aggregates in Escherichia coli, partially associating with mobile foci of IbpA-YFP. BMC Microbiology. 2010;10.

9. Volontè F, Pollegioni L, Molla G, Frattini L, Marinelli F, Piubelli L. Production of recombinant cholesterol oxidase containing covalently bound FAD in Escherichia coli. BMC Biotechnology. 2010;10.

10. Hrmova M, Stone BA, Fincher GB. High-yield production, refolding and a molecular modelling of the catalytic module of (1,3)-β-D-glucan (curdlan) synthase from Agrobacterium sp. Glycoconjugate Journal. 2010;27:461–76.

11. Beassoni PR, Berti FP de, Otero LH, Risso VA, Ferreyra RG, Lisa AT, et al. Preparation and biophysical characterization of recombinant Pseudomonas aeruginosa phosphorylcholine phosphatase. Protein Expression and Purification [Internet]. Elsevier Inc.; 2010;71:153–9. Available from: http://dx.doi.org/10.1016/j.pep.2010.01.006

12. Kanao T, Matsumoto C, Shiraga K, Yoshida K, Takada J, Kamimura K. Recombinant tetrathionate hydrolase from Acidithiobacillus ferrooxidans requires exposure to acidic conditions for proper folding. FEMS Microbiology Letters. 2010;309:43–7.

13. Li S, Pang H, Lin K, Xu J, Zhao J, Fan L. Refolding, purification and characterization of an organic solvent-tolerant lipase from Serratia marcescens ECU1010. Journal of Molecular Catalysis B: Enzymatic [Internet]. Elsevier B.V.; 2011;71:171–6. Available from: http://dx.doi.org/10.1016/j.molcatb.2011.04.016

14. Kumar S, Sharma R, Tewari R. Production of N-Acetylglucosamine Using Recombinant Chitinolytic Enzymes. Indian Journal of Microbiology [Internet]. 2011;51:319–25. Available from: http://link.springer.com/10.1007/s12088-011-0157-7

15. Curiel JA, de las Rivas B, Mancheño JM, Muñoz R. The pURI family of expression vectors: A versatile set of ligation independent cloning plasmids for producing recombinant His-fusion proteins. Protein Expression and Purification [Internet]. 2011;76:44–53. Available from: https://linkinghub.elsevier.com/retrieve/pii/S1046592810003013

16. Ainciart N, Zylberman V, Craig PO, Nygaard D, Bonomi HR, Cauerhff AA, et al. Sensing the dissociation of a polymeric enzyme by means of an engineered intrinsic probe. Proteins: Structure, Function, and Bioinformatics [Internet]. 2011;79:1079–88. Available from: http://doi.wiley.com/10.1002/prot.22945

17. Phillips RS, Ghaffari R, Dinh P, Lima S, Bartlett D. Properties of tryptophan indole-lyase from a piezophilic bacterium, Photobacterium profundum SS9. Archives of Biochemistry and Biophysics [Internet]. Elsevier Inc.; 2011;506:35–41. Available from: http://dx.doi.org/10.1016/j.abb.2010.11.002

18. Tayyab M, Rashid N, Akhtar M. Isolation and identification of lipase producing thermophilic Geobacillus sp. SBS-4S: Cloning and characterization of the lipase. Journal of Bioscience and Bioengineering [Internet]. The Society for Biotechnology, Japan; 2011;111:272–8. Available from: http://dx.doi.org/10.1016/j.jbiosc.2010.11.015

19. Hofer M, Bönsch K, Greiner-Stöffele T, Ballschmiter M. Characterization and Engineering of a Novel Pyrroloquinoline Quinone Dependent Glucose Dehydrogenase from Sorangium cellulosum So ce56. Molecular Biotechnology [Internet]. 2011;47:253–61. Available from: http://link.springer.com/10.1007/s12033-010-9339-5

20. Rajnish KN, Asraf SAKS, Manju N, Gunasekaran P. Functional characterization of a putative β-lactamase gene in the genome of Zymomonas mobilis. Biotechnology Letters [Internet]. 2011;33:2425–30. Available from: http://link.springer.com/10.1007/s10529-011-0704-7

21. Abuhammad A, Lack N, Schweichler J, Staunton D, Sim RB, Sim E. Improvement of the expression and purification of Mycobacterium tuberculosis arylamine N-acetyltransferase (TBNAT) a potential target for novel anti-tubercular agents. Protein Expression and Purification [Internet]. Elsevier Inc.; 2011;80:246–52. Available from: http://dx.doi.org/10.1016/j.pep.2011.06.021

22. Arroyo M, García-Hidalgo J, Villalón M, de Eugenio L, Hormigo D, Acebal C, et al. Characterization of a novel immobilized biocatalyst obtained by matrix-assisted refolding of recombinant polyhydroxyoctanoate depolymerase from Pseudomonas putida KT2442 isolated from inclusion bodies. Journal of Industrial Microbiology and Biotechnology. 2011;38:1203–9.

23. Jung HJ, Kim SK, Min WK, Lee SS, Park K, Park YC, et al. Polycationic amino acid tags enhance soluble expression of Candida antarctica lipase B in recombinant Escherichia coli. Bioprocess and Biosystems Engineering. 2011;34:833–9.

24. Shuo-Shuo C, Xue-Zheng L, Ji-Hong S. Effects of co-expression of molecular chaperones on heterologous soluble expression of the cold-active lipase Lip-948. Protein Expression and Purification [Internet]. Elsevier Inc.; 2011;77:166–72. Available from: http://dx.doi.org/10.1016/j.pep.2011.01.009

25. Sans C, García-Fruitós E, Ferraz RM, González-Montalbán N, Rinas U, López-Santín J, et al. Inclusion bodies of fuculose-1-phosphate aldolase as stable and reusable biocatalysts. Biotechnology Progress [Internet]. 2012;28:421–7. Available from: http://doi.wiley.com/10.1002/btpr.1518

26. Alexander AK, Biedermann D, Fink MJ, Mihovilovic MD, Mattes TE. Enantioselective oxidation by a cyclohexanone monooxygenase from the xenobiotic-degrading Polaromonas sp. strain JS666. Journal of Molecular Catalysis B: Enzymatic [Internet]. Elsevier B.V.; 2012;78:105–10. Available from: http://dx.doi.org/10.1016/j.molcatb.2012.03.002

27. Kim HT, Chung JH, Wang D, Lee J, Woo HC, Choi I-G, et al. Depolymerization of alginate into a monomeric sugar acid using Alg17C, an exo-oligoalginate lyase cloned from Saccharophagus degradans 2-40. Applied Microbiology and Biotechnology [Internet]. 2012;93:2233–9. Available from: http://link.springer.com/10.1007/s00253-012-3882-x

28. Munawar N, Engel PC. Overexpression in a non-native halophilic host and biotechnological potential of NAD+-dependent glutamate dehydrogenase from Halobacterium salinarum strain NRC-36014. Extremophiles [Internet]. 2012;16:463–76. Available from: http://link.springer.com/10.1007/s00792-012-0446-z

29. Toda H, Imae R, Komio T, Itoh N. Expression and characterization of styrene monooxygenases of Rhodococcus sp. ST-5 and ST-10 for synthesizing enantiopure (S)-epoxides. Applied Microbiology and Biotechnology [Internet]. 2012;96:407–18. Available from: http://link.springer.com/10.1007/s00253-011-3849-3

30. Pérez D, Kovačić F, Wilhelm S, Jaeger K-E, García MT, Ventosa A, et al. Identification of amino acids involved in the hydrolytic activity of lipase LipBL from Marinobacter lipolyticus. Microbiology [Internet]. 2012;158:2192–203. Available from: https://www.microbiologyresearch.org/content/journal/micro/10.1099/mic.0.058792-0

31. Vikram S, Pandey J, Bhalla N, Pandey G, Ghosh A, Khan F, et al. Branching of the p-nitrophenol (PNP) degradation pathway in burkholderia sp. Strain SJ98: Evidences from genetic characterization of PNP gene cluster. AMB Express [Internet]. 2012;2:30. Available from: http://amb-express.springeropen.com/articles/10.1186/2191-0855-2-30

32. Upadhyay AK, Murmu A, Singh A, Panda AK. Kinetics of Inclusion Body Formation and Its Correlation with the Characteristics of Protein Aggregates in Escherichia coli. Herman C, editor. PLoS ONE [Internet]. 2012;7:e33951. Available from: https://dx.plos.org/10.1371/journal.pone.0033951

33. Ye W, Liu J, Wang H, Wang J, Wang X. Cloning, expression, purification, and characterization of a glutamate-specific endopeptidase from Bacillus licheniformis. Protein Expression and Purification [Internet]. Elsevier Inc.; 2012;82:138–43. Available from: http://dx.doi.org/10.1016/j.pep.2011.12.001

34. Futakuchi N, Nakatani M, Takahata M, Mitsuyama J. In vitro antichlamydial activity of garenoxacin against Chlamydia trachomatis. Journal of Infection and Chemotherapy [Internet]. Elsevier; 2012;18:428–35. Available from: http://dx.doi.org/10.1007/s10156-011-0345-8

35. Okai M, Ohtsuka J, Asano A, Guo L, Miyakawa T, Miyazono KI, et al. High pressure refolding, purification, and crystallization of flavin reductase from Sulfolobus tokodaii strain 7. Protein Expression and Purification [Internet]. Elsevier Inc.; 2012;84:214–8. Available from: http://dx.doi.org/10.1016/j.pep.2012.06.006

36. Lundqvist LCE, Jam M, Barbeyron T, Czjzek M, Sandström C. Substrate specificity of the recombinant alginate lyase from the marine bacteria Pseudomonas alginovora. Carbohydrate Research [Internet]. Elsevier Ltd; 2012;352:44–50. Available from: http://dx.doi.org/10.1016/j.carres.2012.02.014

37. Singh B, Lee CB, Park JW, Sohng JK. The amino acid sequences in the C-terminal region of glucose-1-phosphate thymidylyltransferases determine their soluble expression in Escherichia coli. Protein Engineering, Design and Selection. 2012;25:179–87.

38. Korovashkina AS, Rymko AN, Kvach S v., Zinchenko AI. Enzymatic synthesis of c-di-GMP using inclusion bodies of Thermotoga maritima full-length diguanylate cyclase. Journal of Biotechnology [Internet]. Elsevier B.V.; 2012;164:276–80. Available from: http://dx.doi.org/10.1016/j.jbiotec.2012.12.006

39. Mohammadi HS, Omidinia E. Process integration for the recovery and purification of recombinant Pseudomonas fluorescens proline dehydrogenase using aqueous two-phase systems. Journal of Chromatography B [Internet]. Elsevier B.V.; 2013;929:11–7. Available from: http://dx.doi.org/10.1016/j.jchromb.2013.03.024

40. Kovalenko GA, Beklemishev AB, Perminova L v., Mamaev AL, Rudina NA, Moseenkov SI, et al. Immobilization of recombinant E. coli thermostable lipase by entrapment inside silica xerogel and nanocarbon-in-silica composites. Journal of Molecular Catalysis B: Enzymatic [Internet]. Elsevier B.V.; 2013;98:78–86. Available from: http://dx.doi.org/10.1016/j.molcatb.2013.09.022

41. Chan GF, Rashid NAA, Yusoff ARM. Expression, purification and characterization of flavin reductase from Citrobacter freundii A1. Annals of Microbiology [Internet]. 2013;63:343–51. Available from: http://link.springer.com/10.1007/s13213-012-0480-1

42. Shavandi M, Soheili M, Zareian S, Akbari N, Khajeh K. The gene cloning, overexpression, purification, and characterization of dibenzothiophene monooxygenase and desulfinase from Gordonia alkanivorans RIPI90A. Journal of Petroleum Science and Technology [Internet]. 2013;3:57–64. Available from: http://jpst.ripi.ir/?_action=articleInfo&article=306

43. Chang J, Lee Y-S, Fang S-J, Park I-H, Choi Y-L. Recombinant Expression and Characterization of an Organic-Solvent-Tolerant α-Amylase from Exiguobacterium sp. DAU5. Applied Biochemistry and Biotechnology [Internet]. 2013;169:1870–83. Available from: http://link.springer.com/10.1007/s12010-013-0101-x

44. Kim J, Copley SD. The Orphan Protein Bis-γ-glutamylcystine Reductase Joins the Pyridine Nucleotide Disulfide Reductase Family. Biochemistry [Internet]. 2013;52:2905–13. Available from: https://pubs.acs.org/doi/10.1021/bi4003343

45. Uehara R, Ueda Y, You D, Koga Y, Kanaya S. Accelerated maturation of Tk‐subtilisin by a Leu→ Pro romutation at the C‐terminus of the propeptide, which reduces the binding of the propeptide to Tk‐subtilisin. The FEBS Journal [Internet]. 2013;280:994–1006. Available from: https://onlinelibrary.wiley.com/doi/abs/10.1111/febs.12091

46. Mollania N, Khajeh K, Ranjbar B, Rashno F, Akbari N, Fathi-Roudsari M. An efficient in vitro refolding of recombinant bacterial laccase in Escherichia coli. Enzyme and Microbial Technology [Internet]. Elsevier Inc.; 2013;52:325–30. Available from: http://dx.doi.org/10.1016/j.enzmictec.2013.03.006

47. Kufner K, Lipps G. Construction of a chimeric thermoacidophilic beta-endoglucanase. BMC Biochemistry [Internet]. 2013;14:11. Available from: http://www.biomedcentral.com/1471-2091/14/11

48. Falcioni F, Blank LM, Frick O, Karau A, Bühler B, Schmida A. Proline availability regulates proline-4-hydroxylase synthesis and substrate uptake in proline-hydroxylating recombinant Escherichia coli. Applied and Environmental Microbiology. 2013;79:3091–100.

49. Fayura LR, Boretsky YR, Pynyaha Y v., Wheatley DN, Sibirny AA. Improved method for expression and isolation of the Mycoplasma hominis arginine deiminase from the recombinant strain of Escherichia coli. Journal of Biotechnology [Internet]. Elsevier B.V.; 2013;167:420–6. Available from: http://dx.doi.org/10.1016/j.jbiotec.2013.06.025

50. Zhu S, Gong C, Ren L, Li X, Song D, Zheng G. A simple and effective strategy for solving the problem of inclusion bodies in recombinant protein technology: His-tag deletions enhance soluble expression. Applied Microbiology and Biotechnology. 2013;97:837–45.

51. Liu L, Hossain GS, Shin HD, Li J, Du G, Chen J. One-step production of α-ketoglutaric acid from glutamic acid with an engineered l-amino acid deaminase from Proteus mirabilis. Journal of Biotechnology [Internet]. Elsevier B.V.; 2013;164:97–104. Available from: http://dx.doi.org/10.1016/j.jbiotec.2013.01.005

52. Puspasari F, Radjasa OK, Noer AS, Nurachman Z, Syah YM, van der Maarel M, et al. Raw starch-degrading α-amylase from Bacillus aquimaris MKSC 6.2: Isolation and expression of the gene, bioinformatics and biochemical characterization of the recombinant enzyme. Journal of Applied Microbiology. 2013;114:108–20.

53. Huang Z, Zhang C, Chen S, Ye F, Xing XH. Active inclusion bodies of acid phosphatase PhoC: Aggregation induced by GFP fusion and activities modulated by linker flexibility. Microbial Cell Factories [Internet]. Microbial Cell Factories; 2013;12:1. Available from: Microbial Cell Factories

54. Ageitos JM, Vallejo JA, Serrat M, Sánchez-Pérez Á, Villa TG. In vitro Ca2+-dependent maturation of milk-clotting recombinant epr: Minor extracellular protease: From bacillus licheniformis. Molecular Biotechnology. 2013;54:304–11.

55. Storm P, Tibiletti T, Hall M, Funk C. Refolding and Enzyme Kinetic Studies on the Ferrochelatase of the Cyanobacterium Synechocystis sp. PCC 6803. PLoS ONE. 2013;8.

56. Novototskaya-Vlasova K, Petrovskaya L, Kryukova E, Rivkina E, Dolgikh D, Kirpichnikov M. Expression and chaperone-assisted refolding of a new cold-active lipase from Psychrobacter cryohalolentis K5T. Protein Expression and Purification [Internet]. Elsevier Inc.; 2013;91:96–103. Available from: http://dx.doi.org/10.1016/j.pep.2013.07.011

57. Wu H, Yu X, Chen L, Wu G. Cloning, overexpression and characterization of a thermostable pullulanase from Thermus thermophilus HB27. Protein Expression and Purification [Internet]. Elsevier Inc.; 2014;95:22–7. Available from: http://dx.doi.org/10.1016/j.pep.2013.11.010

58. Cai X, Ma J, Wei D, Lin J, Wei W. Functional expression of a novel alkaline-adapted lipase of Bacillus amyloliquefaciens from stinky tofu brine and development of immobilized enzyme for biodiesel production. Antonie van Leeuwenhoek [Internet]. 2014;106:1049–60. Available from: http://link.springer.com/10.1007/s10482-014-0274-5

59. Li L, Wang P, Tang Y. C-glycosylation of anhydrotetracycline scaffold with SsfS6 from the SF2575 biosynthetic pathway. The Journal of Antibiotics [Internet]. 2014;67:65–70. Available from: http://www.nature.com/articles/ja201388

60. Singh G, Arya S, Narang D, Jadeja D, Singh G, Gupta UD, et al. Characterization of an acid inducible lipase Rv3203 from Mycobacterium tuberculosis H37Rv. Molecular Biology Reports [Internet]. 2014;41:285–96. Available from: http://link.springer.com/10.1007/s11033-013-2861-3

61. Gröning JAD, Kaschabek SR, Schlömann M, Tischler D. A mechanistic study on SMOB-ADP1: an NADH:flavin oxidoreductase of the two-component styrene monooxygenase of Acinetobacter baylyi ADP1. Archives of Microbiology [Internet]. 2014;196:829–45. Available from: http://link.springer.com/10.1007/s00203-014-1022-y

62. Allen KD, Wang SC. Initial characterization of Fom3 from Streptomyces wedmorensis: The methyltransferase in fosfomycin biosynthesis. Archives of Biochemistry and Biophysics [Internet]. Elsevier Inc.; 2014;543:67–73. Available from: http://dx.doi.org/10.1016/j.abb.2013.12.004

63. Gröning JAD, Eulberg D, Tischler D, Kaschabek SR, Schlömann M. Gene redundancy of two-component (chloro)phenol hydroxylases in Rhodococcus opacus 1CP. FEMS Microbiology Letters [Internet]. 2014;361:68–75. Available from: https://academic.oup.com/femsle/article-lookup/doi/10.1111/1574-6968.12616

64. Chen A, Li Y, Liu X, Long Q, Yang Y, Bai Z. Soluble expression of pullulanase from Bacillus acidopullulyticus in Escherichia coli by tightly controlling basal expression. Journal of Industrial Microbiology & Biotechnology [Internet]. 2014;41:1803–10. Available from: http://link.springer.com/10.1007/s10295-014-1523-3

65. Jia D, Yang Y, Peng Z, Zhang D, Li J, Liu L, et al. High Efficiency Preparation and Characterization of Intact Poly(Vinyl Alcohol) Dehydrogenase from Sphingopyxis sp.113P3 in Escherichia coli by Inclusion Bodies Renaturation. Applied Biochemistry and Biotechnology [Internet]. 2014;172:2540–51. Available from: http://link.springer.com/10.1007/s12010-013-0703-3

66. Wang Y, Li Y-Z. Cultivation to improve in vivo solubility of overexpressed arginine deiminases in Escherichia coliand the enzyme characteristics. BMC Biotechnology [Internet]. 2014;14:53. Available from: https://bmcbiotechnol.biomedcentral.com/articles/10.1186/1472-6750-14-53

67. Nguyen SLT, Quyen DT, Vu HD. Highly effective renaturation of a streptokinase from streptococcus pyogenes DT7 as inclusion bodies overexpressed in escherichia coli. BioMed Research International. Hindawi Publishing Corporation; 2014;2014.

68. Liu Y, Cui W, Liu Z, Cui Y, Xia Y, Kobayashi M, et al. Enhancement of thermo-stability and product tolerance of Pseudomonas putida nitrile hydratase by fusing with self-assembling peptide. Journal of Bioscience and Bioengineering [Internet]. Elsevier Ltd; 2014;118:249–52. Available from: http://dx.doi.org/10.1016/j.jbiosc.2014.02.015

69. Dong Q, Yan X, Zheng M, Yang Z. Characterization of an extremely thermostable but cold-adaptive β-galactosidase from the hyperthermophilic archaeon Pyrococcus furiosus for use as a recombinant aggregation for batch lactose degradation at high temperature. Journal of Bioscience and Bioengineering [Internet]. Elsevier Ltd; 2014;117:706–10. Available from: http://dx.doi.org/10.1016/j.jbiosc.2013.12.002

70. Li Z, Wei P, Cheng H, He P, Wang Q, Jiang N. Functional role of β domain in the Thermoanaerobacter tengcongensis glucoamylase. Applied Microbiology and Biotechnology. 2014;98:2091–9.

71. Ninh PH, Honda K, Sakai T, Okano K, Ohtake H. Assembly and multiple gene expression of thermophilic enzymes in Escherichia coli for in vitro metabolic engineering. Biotechnology and Bioengineering [Internet]. 2015;112:189–96. Available from: http://doi.wiley.com/10.1002/bit.25338

72. Salis B, Spinetti G, Scaramuzza S, Bossi M, Saccani Jotti G, Tonon G, et al. High-level expression of a recombinant active microbial transglutaminase in Escherichia coli. BMC Biotechnology [Internet]. BMC Biotechnology; 2015;15:84. Available from: http://dx.doi.org/10.1186/s12896-015-0202-4

73. Elleuche S, Qoura FM, Lorenz U, Rehn T, Brück T, Antranikian G. Cloning, expression and characterization of the recombinant cold-active type-I pullulanase from Shewanella arctica. Journal of Molecular Catalysis B: Enzymatic [Internet]. Elsevier B.V.; 2015;116:70–7. Available from: http://dx.doi.org/10.1016/j.molcatb.2015.03.001

74. Schröder C, Blank S, Antranikian G. First Glycoside Hydrolase Family 2 Enzymes from Thermus antranikianii and Thermus brockianus with β-Glucosidase Activity. Frontiers in Bioengineering and Biotechnology [Internet]. 2015;3:1–10. Available from: http://journal.frontiersin.org/Article/10.3389/fbioe.2015.00076/abstract

75. Chacón-Verdú MD, Campillo-Brocal JC, Lucas-Elío P, Davidson VL, Sánchez-Amat A. Characterization of recombinant biosynthetic precursors of the cysteine tryptophylquinone cofactors of l-lysine-epsilon-oxidase and glycine oxidase from Marinomonas mediterranea. Biochimica et Biophysica Acta (BBA) - Proteins and Proteomics [Internet]. Elsevier B.V.; 2015;1854:1123–31. Available from: http://dx.doi.org/10.1016/j.bbapap.2014.12.018

76. Akparov VK, Timofeev VI, Khaliullin IG, Švedas V, Chestukhina GG, Kuranova IP. Structural insights into the broad substrate specificity of carboxypeptidase T from Thermoactinomyces vulgaris. FEBS Journal [Internet]. 2015;282:1214–24. Available from: http://doi.wiley.com/10.1111/febs.13210

77. Odunuga OO, Adekoya OA, Sylte I. High-level expression of pseudolysin, the extracellular elastase of Pseudomonas aeruginosa, in Escherichia coli and its purification. Protein Expression and Purification [Internet]. Elsevier Inc.; 2015;113:79–84. Available from: http://dx.doi.org/10.1016/j.pep.2015.05.005

78. Pei X, Wang Q, Meng L, Li J, Yang Z, Yin X, et al. Chaperones-assisted soluble expression and maturation of recombinant Co-type nitrile hydratase in Escherichia coli to avoid the need for a low induction temperature. Journal of Biotechnology [Internet]. Elsevier B.V.; 2015;203:9–16. Available from: http://dx.doi.org/10.1016/j.jbiotec.2015.03.004

79. Kumar S, Jain KK, Singh A, Panda AK, Kuhad RC. Characterization of recombinant pectate lyase refolded from inclusion bodies generated in E. coli BL21(DE3). Protein Expression and Purification [Internet]. Elsevier Inc.; 2015;110:43–51. Available from: http://dx.doi.org/10.1016/j.pep.2014.12.003

80. Klermund L, Riederer A, Groher A, Castiglione K. High-level soluble expression of a bacterial N-acyl-d-glucosamine 2-epimerase in recombinant Escherichia coli. Protein Expression and Purification [Internet]. Elsevier Inc.; 2015;111:36–41. Available from: http://dx.doi.org/10.1016/j.pep.2015.03.009

81. Doukyu N, Nihei S. Cholesterol oxidase with high catalytic activity from Pseudomonas aeruginosa: Screening, molecular genetic analysis, expression and characterization. Journal of Bioscience and Bioengineering [Internet]. Elsevier Ltd; 2015;120:24–30. Available from: http://dx.doi.org/10.1016/j.jbiosc.2014.12.003

82. García-Fraga B, da Silva AF, López-Seijas J, Sieiro C. Optimized expression conditions for enhancing production of two recombinant chitinolytic enzymes from different prokaryote domains. Bioprocess and Biosystems Engineering. 2015;38:2477–86.

83. Yang W, Cao H, Xu L, Zhang H, Yan Y. A novel eurythermic and thermostale lipase LipM from Pseudomonas moraviensis M9 and its application in the partial hydrolysis of algal oil. BMC Biotechnology. 2015;15:1–15.

84. Xin Y, Zheng M, Wang Q, Lu L, Zhang L, Tong Y, et al. Structural and catalytic alteration of sarcosine oxidase through reconstruction with coenzyme-like ligands. Journal of Molecular Catalysis B: Enzymatic [Internet]. Elsevier B.V.; 2016;133:S250–8. Available from: https://doi.org/10.1016/j.molcatb.2017.01.011

85. Dong Q, Ruan L, Shi H. A β-agarase with high pH stability from Flammeovirga sp. SJP92. Carbohydrate Research [Internet]. Elsevier Ltd; 2016;432:1–8. Available from: http://dx.doi.org/10.1016/j.carres.2016.05.002

86. Kim H, Yeon YJ, Choi YR, Song W, Pack SP, Choi YS. A cold-adapted tyrosinase with an abnormally high monophenolase/diphenolase activity ratio originating from the marine archaeon Candidatus Nitrosopumilus koreensis. Biotechnology Letters [Internet]. Springer Netherlands; 2016;38:1535–42. Available from: http://link.springer.com/10.1007/s10529-016-2125-0

87. Garg R, Srivastava R, Brahma V, Verma L, Karthikeyan S, Sahni G. Biochemical and structural characterization of a novel halotolerant cellulase from soil metagenome. Scientific Reports [Internet]. Nature Publishing Group; 2016;6:39634. Available from: http://www.nature.com/articles/srep39634

88. Blaszczyk AJ, Silakov A, Zhang B, Maiocco SJ, Lanz ND, Kelly WL, et al. Spectroscopic and Electrochemical Characterization of the Iron–Sulfur and Cobalamin Cofactors of TsrM, an Unusual Radical S -Adenosylmethionine Methylase. Journal of the American Chemical Society [Internet]. 2016;138:3416–26. Available from: https://pubs.acs.org/doi/10.1021/jacs.5b12592

89. Dang G, Cao J, Cui Y, Song N, Chen L, Pang H, et al. Characterization of Rv0888, a Novel Extracellular Nuclease from Mycobacterium tuberculosis. Scientific Reports [Internet]. Nature Publishing Group; 2016;6:19033. Available from: http://www.nature.com/articles/srep19033

90. Chen K, Wu S, Zhu L, Zhang C, Xiang W, Deng Z, et al. Substitution of a Single Amino Acid Reverses the Regiospecificity of the Baeyer–Villiger Monooxygenase PntE in the Biosynthesis of the Antibiotic Pentalenolactone. Biochemistry [Internet]. 2016;55:6696–704. Available from: https://pubs.acs.org/doi/10.1021/acs.biochem.6b01040

91. Ni H, Guo P-C, Jiang W-L, Fan X-M, Luo X-Y, Li H-H. Expression of nattokinase in Escherichia coli and renaturation of its inclusion body. Journal of Biotechnology [Internet]. 2016;231:65–71. Available from: https://linkinghub.elsevier.com/retrieve/pii/S0168165616302905

92. Tong Y, Feng S, Xin Y, Yang H, Zhang L, Wang W, et al. Enhancement of soluble expression of codon-optimized Thermomicrobium roseum sarcosine oxidase in Escherichia coli via chaperone co-expression. Journal of Biotechnology. Elsevier B.V.; 2016;218:75–84.

93. Zhao M, Cai M, Wu F, Zhang Y, Xiong Z, Xu P. Recombinant expression, refolding, purification and characterization of Pseudomonas aeruginosa protease IV in Escherichia coli. Protein Expression and Purification [Internet]. Elsevier Ltd; 2016;126:69–76. Available from: http://dx.doi.org/10.1016/j.pep.2016.05.019

94. Spieler V, Valldorf B, Maaß F, Kleinschek A, Hüttenhain SH, Kolmar H. Coupled reactions on bioparticles: Stereoselective reduction with cofactor regeneration on PhaC inclusion bodies. Biotechnology Journal [Internet]. 2016;11:890–8. Available from: http://doi.wiley.com/10.1002/biot.201500495

95. Dangi AK, Rishi P, Tewari R. Enhancing the Yield of Active Recombinant Chitobiase by Physico-Chemical and In Vitro Refolding Studies. Protein Journal. Springer New York LLC; 2016;35:72–9.

96. Ganasen M, Yaacob N, Rahman RNZRA, Leow ATC, Basri M, Salleh AB, et al. Cold-adapted organic solvent tolerant alkalophilic family I.3 lipase from an Antarctic Pseudomonas. International Journal of Biological Macromolecules [Internet]. Elsevier B.V.; 2016;92:1266–76. Available from: http://dx.doi.org/10.1016/j.ijbiomac.2016.06.095

97. Peng S, Chu Z, Lu J, Li D, Wang Y, Yang S, et al. Co-expression of chaperones from P. furiosus enhanced the soluble expression of the recombinant hyperthermophilic α-amylase in E. coli. Cell Stress and Chaperones. 2016;21:477–84.

98. Wang CH, Zhang C, Xing XH. Metabolic engineering of Escherichia coli cell factory for highly active xanthine dehydrogenase production. Bioresource Technology [Internet]. Elsevier Ltd; 2017;245:1782–9. Available from: https://doi.org/10.1016/j.biortech.2017.05.144

99. Kim S, Park H, Choi J. Cloning and Characterization of Cold-Adapted α-Amylase from Antarctic Arthrobacter agilis. Applied Biochemistry and Biotechnology [Internet]. Applied Biochemistry and Biotechnology; 2017;181:1048–59. Available from: http://link.springer.com/10.1007/s12010-016-2267-5

100. Cheng H, Luo Z, Lu M, Gao S, Wang S. The hyperthermophilic α-amylase from Thermococcus sp. HJ21 does not require exogenous calcium for thermostability because of high-binding affinity to calcium. Journal of Microbiology [Internet]. 2017;55:379–87. Available from: http://link.springer.com/10.1007/s12275-017-6416-5

101. Zhang Y, Wang H, Wang X, Hu B, Zhang C, Jin W, et al. Identification of the key amino acid sites of the carbendazim hydrolase (MheI) from a novel carbendazim-degrading strain Mycobacterium sp. SD-4. Journal of Hazardous Materials [Internet]. 2017;331:55–62. Available from: https://linkinghub.elsevier.com/retrieve/pii/S0304389417300882

102. Guan L, Chen L, Chen Y, Zhang N, Han Y. Expression and Activity Analysis of Fructosyltransferase from Aspergillus oryzae. Protein Journal. Springer US; 2017;36:352–60.

103. Shao M, Chen Y, Zhang X, Rao Z, Xu M, Yang T, et al. Enhanced intracellular soluble production of 3-ketosteroid-Δ1-dehydrogenase from Mycobacterium neoaurum in Escherichia coli and its application in the androst-1,4-diene-3,17-dione production. Journal of Chemical Technology and Biotechnology. 2017;92:350–7.

104. Li Y, Zhou Z, Chen Z. High-level production of ChSase ABC I by co-expressing molecular chaperones in Escherichia coli. International Journal of Biological Macromolecules [Internet]. 2018;119:779–84. Available from: https://linkinghub.elsevier.com/retrieve/pii/S0141813017349218

105. Bakholdina SI, Sidorin E v., Khomenko VA, Isaeva MP, Kim NY, Bystritskaya EP, et al. The Effect of Conditions of the Expression of the Recombinant Outer Membrane Phospholipase А1 from Yersinia pseudotuberculosis on the Structure and Properties of Inclusion Bodies. Russian Journal of Bioorganic Chemistry [Internet]. 2018;44:178–87. Available from: http://link.springer.com/10.1134/S1068162018020061

106. Kim KH, Jia X, Jia B, Jeon CO. Identification and Characterization of L-Malate Dehydrogenases and the L-Lactate-Biosynthetic Pathway in Leuconostoc mesenteroides ATCC 8293. Journal of Agricultural and Food Chemistry [Internet]. 2018;66:8086–93. Available from: https://pubs.acs.org/doi/10.1021/acs.jafc.8b02649

107. Yamamoto T, Ugai H, Nakayama-Imaohji H, Tada A, Elahi M, Houchi H, et al. Characterization of a recombinant Bacteroides fragilis sialidase expressed in Escherichia coli. Anaerobe [Internet]. Elsevier Ltd; 2018;50:69–75. Available from: https://doi.org/10.1016/j.anaerobe.2018.02.003

108. Saeed H, Ali H, Soudan H, Embaby A, El-Sharkawy A, Farag A, et al. Molecular cloning, structural modeling and production of recombinant Aspergillus terreus L. asparaginase in Escherichia coli. International Journal of Biological Macromolecules [Internet]. Elsevier B.V.; 2018;106:1041–51. Available from: https://doi.org/10.1016/j.ijbiomac.2017.08.110

109. Luo Y, Zhao Q, Liu Q, Feng Y. An Artificial Biosynthetic Pathway for 2-Amino-1,3-Propanediol Production Using Metabolically Engineered Escherichia coli. ACS Synthetic Biology [Internet]. 2019;8:548–56. Available from: https://pubs.acs.org/doi/10.1021/acssynbio.8b00466

110. Jaroensuk J, Intasian P, Kiattisewee C, Munkajohnpon P, Chunthaboon P, Buttranon S, et al. Addition of formate dehydrogenase increases the production of renewable alkane from an engineered metabolic pathway. Journal of Biological Chemistry [Internet]. 2019;294:11536–48. Available from: http://www.jbc.org/lookup/doi/10.1074/jbc.RA119.008246

111. de Almeida TP, van Schie MMCH, Ma A, Tieves F, Younes SHH, Fernández-Fueyo E, et al. Efficient Aerobic Oxidation of trans -2-Hexen-1-ol using the Aryl Alcohol Oxidase from Pleurotus eryngii. Advanced Synthesis & Catalysis [Internet]. 2019;361:2668–72. Available from: http://doi.wiley.com/10.1002/adsc.201801312

112. Zhang J, Lu J, Su E. Soluble recombinant pyruvate oxidase production in Escherichia coli can be enhanced and inclusion bodies minimized by avoiding pH stress. Journal of Chemical Technology & Biotechnology [Internet]. 2019;94:2661–70. Available from: https://onlinelibrary.wiley.com/doi/abs/10.1002/jctb.6075

113. Su L, Wu S, Feng J, Wu J. High-efficiency expression of Sulfolobus acidocaldarius maltooligosyl trehalose trehalohydrolase in Escherichia coli through host strain and induction strategy optimization. Bioprocess and Biosystems Engineering [Internet]. Springer Berlin Heidelberg; 2019;42:345–54. Available from: http://dx.doi.org/10.1007/s00449-018-2039-4

114. Flores SS, Nolan V, Perillo MA, Sánchez JM. Superactive β-galactosidase inclusion bodies. Colloids and Surfaces B: Biointerfaces [Internet]. Elsevier; 2019;173:769–75. Available from: https://doi.org/10.1016/j.colsurfb.2018.10.049

115. Srivastava V, Mishra S, Chaudhuri TK. Enhanced production of recombinant serratiopeptidase in Escherichia coli and its characterization as a potential biosimilar to native biotherapeutic counterpart. Microbial Cell Factories [Internet]. BioMed Central; 2019;18:215. Available from: https://doi.org/10.1186/s12934-019-1267-x

116. Mestrom L, Marsden SR, Dieters M, Achterberg P, Stolk L, Bento I, et al. Artificial Fusion of mCherry Enhances Trehalose Transferase Solubility and Stability. Kivisaar M, editor. Applied and Environmental Microbiology [Internet]. 2019;85:1–15. Available from: http://aem.asm.org/lookup/doi/10.1128/AEM.03084-18

117. Shaeer A, Aslam M, Rashid N. A highly stable manganese catalase from Geobacillus thermopakistaniensis: molecular cloning and characterization. Extremophiles [Internet]. Springer Japan; 2019;23:707–18. Available from: https://doi.org/10.1007/s00792-019-01124-5

118. Xie G, Shao Z, Zong L, Li X, Cong D, Huo R. Heterologous expression and characterization of a novel subtilisin-like protease from a thermophilic Thermus thermophilus HB8. International Journal of Biological Macromolecules [Internet]. Elsevier B.V.; 2019;138:528–35. Available from: https://doi.org/10.1016/j.ijbiomac.2019.07.101

119. Ahlawat S, Singh D, Virdi JS, Sharma KK. Molecular modeling and MD-simulation studies: Fast and reliable tool to study the role of low-redox bacterial laccases in the decolorization of various commercial dyes. Environmental Pollution [Internet]. Elsevier Ltd; 2019;253:1056–65. Available from: https://doi.org/10.1016/j.envpol.2019.07.083

120. Shao H, Hu X, Sun L, Zhou W. Gene cloning, expression in E. coli, and in vitro refolding of a lipase from Proteus sp. NH 2-2 and its application for biodiesel production. Biotechnology Letters [Internet]. Springer Netherlands; 2019;41:159–69. Available from: https://doi.org/10.1007/s10529-018-2625-1

121. Ni W, Liu H, Wang P, Wang L, Sun X, Wang H, et al. Evaluation of multiple fused partners on enhancing soluble level of prenyltransferase NovQ in Escherichia coli. Bioprocess and Biosystems Engineering [Internet]. Springer Berlin Heidelberg; 2019;42:465–74. Available from: http://dx.doi.org/10.1007/s00449-018-2050-9

122. Harada K, Nambu Y, Mizuno S, Tsuge T. In vivo and in vitro characterization of hydrophilic protein tag-fused Ralstonia eutropha polyhydroxyalkanoate synthase. International Journal of Biological Macromolecules [Internet]. Elsevier B.V.; 2019;138:379–85. Available from: https://doi.org/10.1016/j.ijbiomac.2019.07.095

123. Kinkar E, Kinkar A, Saleh M. The multicopper oxidase of Mycobacterium tuberculosis (MmcO) exhibits ferroxidase activity and scavenges reactive oxygen species in activated THP-1 cells. International Journal of Medical Microbiology [Internet]. Elsevier; 2019;309:151324. Available from: https://doi.org/10.1016/j.ijmm.2019.06.004

124. Basheer S, Rashid N, Akram MS, Akhtar M. A highly stable laccase from Bacillus subtilis strain R5: Gene cloning and characterization. Bioscience, Biotechnology and Biochemistry [Internet]. Taylor & Francis; 2019;83:436–45. Available from: https://doi.org/10.1080/09168451.2018.1530097

125. Zhang G, An Y, Parvez A, Zabed HM, Yun J, Qi X. Exploring a Highly D-Galactose Specific L-Arabinose Isomerase From Bifidobacterium adolescentis for D-Tagatose Production. Frontiers in Bioengineering and Biotechnology [Internet]. 2020;8:1–10. Available from: https://www.frontiersin.org/article/10.3389/fbioe.2020.00377/full

126. Shah S, Sunder AV, Singh P, Wangikar PP. Characterization and Application of a Robust Glucose Dehydrogenase from Paenibacillus pini for Cofactor Regeneration in Biocatalysis. Indian Journal of Microbiology [Internet]. Springer India; 2020;60:87–95. Available from: https://doi.org/10.1007/s12088-019-00834-w

127. Yao D, Fan J, Han R, Xiao J, Li Q, Xu G, et al. Enhancing soluble expression of sucrose phosphorylase in Escherichia coli by molecular chaperones. Protein Expression and Purification [Internet]. 2020;169:105571. Available from: https://linkinghub.elsevier.com/retrieve/pii/S1046592819305212

128. Zakalskiy AE, Stasyuk NY, Zakalska OM, Boretsky YR, Gonchar M v. Overexpression and one‐step renaturation‐purification of the tagged creatinine deiminase of Corynebacterium glutamicum in Escherichia coli cells. Cell Biology International [Internet]. John Wiley & Sons, Ltd; 2020;44:1204–11. Available from: http://dx.doi.org/10.1002/cbin.11320

129. Jeon SJ, Park JH. Refolding, characterization, and dye decolorization ability of a highly thermostable laccase from Geobacillus sp. JS12. Protein Expression and Purification [Internet]. Elsevier; 2020;173:105646. Available from: https://doi.org/10.1016/j.pep.2020.105646

130. Ge Y, Guo S, Liu T, Zhao C, Li D, Liu Y, et al. Optimizing a production strategy for a nonspecific nuclease from Yersinia enterocolitica subsp. palearctica in genetically engineered Escherichia coli. FEMS Microbiology Letters. Oxford University Press; 2020;366:1–7.

131. Karakaş F, Arslanoğlu A. Gene cloning, heterologous expression, and partial characterization of a novel cold-adapted subfamily I.3 lipase from Pseudomonas fluorescence KE38. Scientific Reports [Internet]. Nature Publishing Group UK; 2020;10:1–13. Available from: https://doi.org/10.1038/s41598-020-79199-w

132. Effendi SSW, Tan SI, Ting WW, Ng IS. Genetic design of co-expressed Mesorhizobium loti carbonic anhydrase and chaperone GroELS to enhancing carbon dioxide sequestration. International Journal of Biological Macromolecules. 2021;167:326–34.

133. Vadala BS, Deshpande S, Apte-Deshpande A. Soluble expression of recombinant active cellulase in E.coli using B.subtilis (natto strain) cellulase gene. Journal of Genetic Engineering and Biotechnology. Journal of Genetic Engineering and Biotechnology; 2021;19.
